# Altered X-chromosome inactivation predisposes to autoimmunity

**DOI:** 10.1126/sciadv.adn6537

**Published:** 2024-05-03

**Authors:** Christophe Huret, Léa Ferrayé, Antoine David, Myriame Mohamed, Nicolas Valentin, Frédéric Charlotte, Magali Savignac, Michele Goodhardt, Jean-Charles Guéry, Claire Rougeulle, Céline Morey

**Affiliations:** ^1^ Université Paris Cité, CNRS, Epigenetics and Cell Fate, F-75013 Paris, France.; ^2^ Toulouse Institute for Infectious and Inflammatory Diseases (Infinity), INSERM UMR1291, CNRS UMR5051, University Paul Sabatier, Toulouse, France.; ^3^ Université Paris Cité, INSERM UMRS 976, Institut de Recherche Saint Louis, F-75010, Paris, France.; ^4^ Université Paris Cité, CNRS, Institut Jacques Monod, F-75013, Paris, France.; ^5^ Sorbonne University, Department of Pathological Anatomy and Cytology, Hôpital Pitié-Salpêtrière Charles Foix, F-75013, Paris, France.

## Abstract

In mammals, males and females show marked differences in immune responses. Males are globally more sensitive to infectious diseases, while females are more susceptible to systemic autoimmunity. X-chromosome inactivation (XCI), the epigenetic mechanism ensuring the silencing of one X in females, may participate in these sex biases. We perturbed the expression of the trigger of XCI, the noncoding RNA *Xist*, in female mice. This resulted in reactivation of genes on the inactive X, including members of the Toll-like receptor 7 (TLR7) signaling pathway, in monocyte/macrophages and dendritic and B cells. Consequently, female mice spontaneously developed inflammatory signs typical of lupus, including anti–nucleic acid autoantibodies, increased frequencies of age-associated and germinal center B cells, and expansion of monocyte/macrophages and dendritic cells. Mechanistically, TLR7 signaling is dysregulated in macrophages, leading to sustained expression of target genes upon stimulation. These findings provide a direct link between maintenance of XCI and female-biased autoimmune manifestations and highlight altered XCI as a cause of autoimmunity.

## Teaser

Perturbation of X-chromosome inactivation, a female-specific process, induces signs of lupus, a disease that mostly affects women.

## INTRODUCTION

In mammals, there is a marked sexual dimorphism in immune responses and functions. This is partly explained by stronger innate and adaptive immunity in adult females, conferring a better response to various types of pathogens and vaccines compared to males. Such enhanced immunity in females may, however, lead to over-reactivity when not properly controlled, hence resulting in autoimmune manifestations. The determinants of this sexual bias are not fully understood. While these differences are partly attributable to sex hormones, X-linked factors represent other likely contributors (*1*–*6*). Men with Klinefelter’s syndrome, who bear an extra X chromosome (47, XXY) in a male hormonal context, have a risk equivalent to women to develop relatively rare immune disorders such as systemic lupus erythematosus (SLE) (*7*), Sjogren’s syndrome (*8*), or Systemic Sclerosis (*9*). Moreover, the female bias in autoimmune diseases such as SLE is observed before puberty (*10*). Incidentally, the X chromosome has a high density of genes involved in immune functions (*11*, *12*), and some of these, including *TLR7*, *TASL*, *CXCR3*, or *CD40LG*, tend to be overexpressed in autoimmune conditions, suggesting a causal link between autoimmunity and X-linked gene regulation (*2*, *5*, *13*). A key role for Toll-like receptor 7 (TLR7), a single-stranded RNA (ssRNA) sensor essential for the defense against RNA viruses that can also be engaged by endogenous ligands, has been established in SLE pathogenesis (*11*). Expression of two copies of *Tlr7* in male mice is sufficient to induce full-blown autoimmunity (*14*, *15*). Recently, a genetic variant of human *TLR7* (*TLR7^Y264H^*, gain of function) has been identified in a young girl with juvenile SLE. Mice carrying this *Tlr7^Y264H^* mutation spontaneously develop a lupus-like disease due to aberrant TLR7 signaling, which results in the accumulation of pathogenic unconventional T-bet^+^ CD11c^+^ memory B cells, also known as age-associated B cells (ABCs) (*16*). Hence, the link between *Tlr7* overexpression and autoimmunity has been firmly established. However, how such *Tlr7* up-regulation is initially triggered, whether perturbation of X-chromosome expression may be involved and, more generally, how broad alteration of X-linked gene expression would affect the fitness of the immune system is unknown.

X-linked gene expression is equalized between the sexes through the transcriptional silencing of most genes of one of the two X chromosomes, at random, in females. This X-chromosome inactivation (XCI) is an essential process established during early embryogenesis and maintained afterward in daughter cells throughout in utero and postnatal life. XCI is triggered by the accumulation of the *Xist* long noncoding RNA (lncRNA) on one of the two X chromosomes [the future inactive X (Xi)]. *Xist* then recruits a series of factors inducing gene silencing in cis (*17*). While, in most cell types, the repressed state is thought to be locked by several layers of chromatin modifications, XCI maintenance in immune cells exhibit certain specific features. First, a number of X-linked genes including *TLR7*, *TASL*, and *CXCR3* tend to escape from XCI and are transcribed from the Xi in a substantial proportion of human immune cells in physiological conditions (*3*, *11*, *18*–*20*). Second, Xi hallmarks including *Xist* lncRNA accumulation and enrichment in repressive histone modifications (notably histone H3 lysine K27 tri-methylation H3K27me3 and histone H2A lysine K119 ubiquitination H2AK119ub) are almost completely lost during B and T lymphopoiesis and regained upon lymphocyte activation (*21*). These marks are either reduced or completely absent in natural killer cells, dendritic cells (DC), and macrophages (MΦ) (*22*). This tendency is exacerbated in B lymphocytes from patients with SLE (*21*) or in stimulated B cells from the lupus mouse model (NZB/W F1) (*23*, *24*). In addition, in human, distinct sets of proteins interact with the *XIST* lncRNA in myeloid compared to lymphoid lineages, suggesting that *XIST* could mediate silencing using different mechanisms depending on the immune cell type (*25*). Third, knocking out (KO) *Xist* when hematopoietic cells are specified during development results in differentiation defects during hematopoiesis, up-regulation of natural XCI escapees (*26*), and aggressive blood cancer in adult mice (*27*). The same *Xist* KO has, however, relatively minor effects when induced in other, non-immune, cell types (*28*, *29*). In human, repressing *XIST* in a B cell line results in increased expression of a subset of X-linked genes that tend to be overexpressed in ABCs of patients with SLE (*25*). Together, these findings suggest that XCI displays specific regulatory requirements in hematopoietic cells. However, whether such a unique XCI plasticity may lead to over-reactivity of female immune system when not properly controlled remains to be formerly addressed (*13*).

**Fig. 1.**
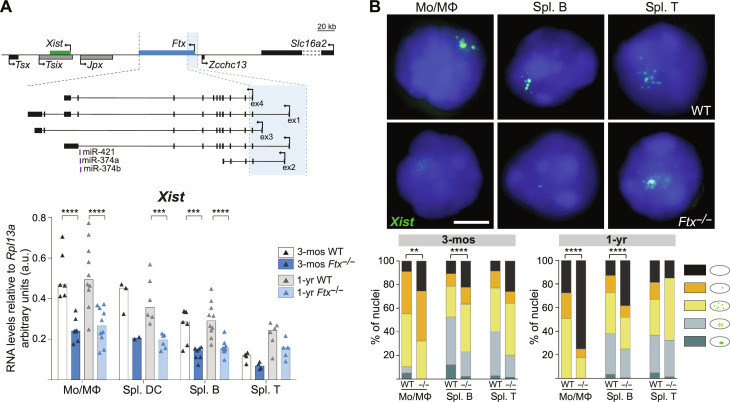
Perturbation of X-inactivation in immune cells of *Ftx^−/−^* females. (**A**) Map of the X inactivation center showing the location of *Xist* (green) and *Ftx* and the *Ftx* promoter region that has been deleted (blue shading). Other noncoding regulators of *Xist* are shown in gray. Underneath, *Xist* RNA levels measured by reverse transcription quantitative polymerase chain reaction (RT-qPCR) in wild-type (WT) and *Ftx^−/−^* females at 3 months (3-mos) and 1-year (1-yr) of age. Monocytes/macrophages (Mo/MΦ) were collected from the bone marrow (BM). Other cell types were collected from the spleen. Each triangle represents RNA levels in a mouse. Bar plots show median values (*t* test, ***P < 0.005 and ****P < 0.001). (**B**) Representative images of RNA fluorescence in situ hybridization (RNA-FISH) for *Xist* (green) on WT and *Ft*x^−/−^ female cells of the indicated cell type. Note that *Xist* lncRNAs tend to be delocalized from the Xi even in WT mice as previously described (*21*). The percentages of cells with different patterns of *Xist* RNA distribution in the cell populations are shown on the histograms (chi-square test, **P < 0.01 and ****P < 0.001; *N* > 2 mice; *n* > 100 nuclei per mice). Scale bar, 5 μm.

To characterize the impact of perturbed X-linked gene regulation on immune functions in vivo, we used the *Ftx* KO background (*Ftx*^−/−^) as a mean to mildly impair XCI while bypassing lethality associated with *Xist* deficiency. This mutation also mimics XCI alterations that are susceptible to occur in vivo. *Ftx* is a noncoding gene of the X-inactivation center (Fig. 1A), which acts as a cis-activator of *Xist* during the initiation of XCI (*30*, *31*). Consequently, deletion of the *Ftx* promoter results in reduced *Xist* expression, impaired accumulation of *Xist* lncRNAs on the Xi, and incomplete X-linked silencing during mouse ES cell differentiation (*32*) as well as during female development (*33*, *34*). Here, we show that, in immune cells of *Ftx*^−/−^ females, XCI is progressively destabilized, resulting in the erosion of silencing of selected X-linked genes with immune functions. These include genes of the TLR7 pathway for which escape from XCI is enhanced. This occurs coincidently with the development of autoimmune manifestations such as splenomegaly, higher percentages of activated T and B lymphocytes, and higher levels of immunoglobulins and of autoantibodies against nucleic acids (NAs) and ribonucleoprotein (RNP) complexes in the serum. Autoantibody production is furthermore associated with the accumulation of CD11c^+^ ABCs and germinal center (GC) B cells in these mice. Mechanistically, MΦ of 1-year-old *Ftx*^−/−^ females exhibit sustained pro-inflammatory cytokine expression upon exogenous activation of the TLR7 pathway, suggesting that *Tlr7* enhanced escape from XCI perpetuates an over-reactive immune environment. Together, these observations provide a direct link between XCI deregulation—a female-specific biological process—and changes in immune cell features and point to alteration in XCI maintenance as a potential trigger of various forms of female-biased autoimmune manifestations.

## RESULTS

### *Ftx* deletion induces aberrant *Xist* expression profiles in immune cells of adult females

To study the effect of XCI perturbation on immune functions during adult life, we created a mouse line carrying a deletion of *Ftx* transcription start sites similar to the mutations generated previously (Fig. 1A) (*32*, *34*). *Ftx* KO animals were born in Mendelian ratios (table S1). Male and females developed normally, appeared healthy overall and fertile with no difference in life span compared to wild-type (WT) animals, as reported in (*33*, *34*). As expected, *Ftx* expression was completely abolished in immune cells of *Ftx*^−/−^ females as measured by reverse transcription quantitative polymerase chain reaction (RT-qPCR) (fig. S1A) and by RNA fluorescence in situ hybridization (RNA-FISH) (fig. S1B). *Xist* expression appeared to be significantly reduced in most *Ftx*^−/−^ immune cells (half the levels of WT) (Fig. 1A), and *Xist* lncRNAs hardly clustered on the Xi in immune cell nuclei from 3 months of age onward (Fig. 1B). This indicates that *Ftx* deletion affects *Xist* expression in adult immune cells. This perturbation can be considered as mild because it does not lead to a loss of *Xist* lncRNAs in all the cells (Fig. 1B).

### Altered *Xist* expression results in overexpression and reactivation of genes on the Xi

**Fig. 2.**
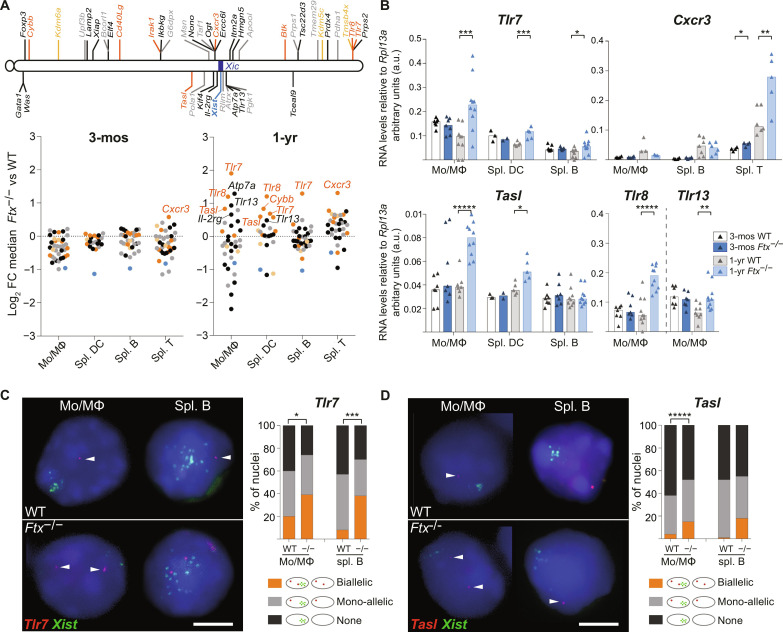
Aberrant X-inactivation results in overexpression of several X-linked genes associated with autoimmune conditions. (**A**) Map of the X chromosome showing genes known to constitutively escape from XCI (yellow), genes with immune function and a tendency to escape from XCI (orange), other genes with immune function (black), and housekeeping genes (gray). Underneath, log_2_ fold change (FC) between median RNA levels as measured by RT-qPCR in KO versus WT mice for each gene, in the indicated cell type, either in 3-month-old (left) or in 1-year-old mice (right). Each dot represents an X-linked gene. The names of genes showing significantly different RNA levels between KO versus WT are indicated on the graphs (*t* test, *P* < 0.05; *n* ≥ 3 mice per genotype). (**B**) RNA levels of *Tlr7*, *Cxcr3*, *Tasl*, *Tlr8*, and *Tlr13* as measured by RT-qPCR in cell types showing detectable expression. Each triangle represents RNA levels in a mouse. Bar plots show median values (*t* test, *P < 0.05, **P < 0.01, ***P < 0.005, and *****P < 0.001). (**C**) Representative images of RNA-FISH for *Tlr7* (red) and *Xist* (green) on WT and *Ftx*^−/−^ cells from 1-year-old female mice. The percentages of cells with biallelic, mono-allelic, or no signals are shown on the histogram (chi-square test, *P < 0.05 and ***P < 0.005; *N* > 2 mice; *n* > 100 nuclei per mice). Scale bar, 5 μm. (**D**) Same as (C) for *Tasl* transcription (chi-square test, *****P < 0.001; *N* > 2 mice; *n* > 100 nuclei per mice).

To determine whether and how X-linked gene silencing is changed upon *Xist* perturbation, we measured the RNA levels of a battery of X-linked genes. We chose genes with different functions and XCI features including immune-related and unrelated genes, genes known to be associated with autoimmune phenotypes, housekeeping genes, and genes known to escape from XCI (Fig. 2A and fig. S2). We used RT-qPCR on a selection of immune cell types from 3-month- and 1-year-old females to accurately quantify the expression levels (Fig. 2A). At 3 months of age, no significant variation of X-linked RNA levels was observed between WT and mutant cells with the exception of *Cxcr3* in T cells (Fig. 2, A and B). In contrast, in 1-year-old females, a number of genes were significantly overexpressed in immune cells of *Ftx*^−/−^ versus WT mice (Fig. 2A). Many of these genes (*Tlr7*, *Tlr8*, *Tasl/CXorf21*, *Cybb*, and *Cxcr3*) naturally escape from XCI in a significant proportion of immune cells (*35*). Examination of RNA levels in individual mice showed high animal-to-animal variability (Fig. 2B), which may reflect variable frequencies of cells in which XCI was not properly established in the hematopoietic stem population.

Higher mRNA levels may result from higher expression of alleles on the active X, from increased expression of alleles that already escaped from XCI in these cells, or from reactivation of Xi alleles in additional cells, which means, in this latter configuration, a relaxation of XCI-mediated silencing of these genes. To discriminate between these possibilities, we performed double RNA-FISH for *Tlr7* and *Xist* or for *Tasl* and *Xist* on monocyte/macrophages (Mo/MΦ) from the bone marrow (BM) of 1-year-old females, in which the differential of expression in *Ftx*^−/−^ versus WT by RT-qPCR was the most pronounced (Fig. 2B). We detected significantly higher percentages of nuclei with two pinpoint signals indicating biallelic expression in *Ftx*^−/−^ compared to WT cells (Fig. 2, C and D). This shows that *Tlr7* or *Tasl* mRNA overexpression in 1-year-old *Ftx*^−/−^ Mo/MΦ results from an increase of the proportion of cells in which these genes escape from XCI in the population compared to WT Mo/MΦ population. Similar increase in the frequency of biallelically expressing cells was also observed for *Tlr7* in B lymphocytes from 1-year-old *Ftx*^−/−^ mice (Fig. 2C).

Not all known escapees appeared overexpressed (fig. S2A), and some genes supposedly subject to XCI (*Tlr13*, *Il-2rg*, and *Atp7a*) showed higher RNA levels in *Ftx*^−/−^ compared to WT cells (Fig. 2, A and B, and fig. S2B). This suggests that labile expression from the Xi may facilitate, but is not a prerequisite for, overexpression and that genes may escape from XCI upon *Xist* perturbation specifically. In contrast, expression of X-linked housekeeping genes was not significantly perturbed in *Ftx*^−/−^ immune cells (fig. S2C). We also observed some genes with lower expression in *Ftx*^−/−^ cells compared to WT (Fig. 2A and fig. S2D) that may constitute secondary targets of X-linked gene overexpression.

Together, these results indicate that impaired *Xist* expression in *Ftx*^−/−^ immune cells does not lead to global reactivation of the Xi. Rather, it appears to induce or enhance escape from XCI of specific X-linked genes, leading to increased expression levels of those genes as time goes by.

### Specific X-linked factors involved in autoimmunity tend to be overexpressed upon XCI perturbation

The most notable feature of X-linked genes affected by *Xist* perturbation is the high number of genes involved in innate immune response (*TLR7*, *TLR8*, *TLR13*, *TASL*, and *CXCR3*), which have been reported to be associated with or to have a causative role in different autoimmune diseases (table S2). They include many endosomal TLRs (TLR7, TLR8, and TLR13) or plasma membrane chemokine receptor (CXCR3). No significant deregulation of two other membrane receptor genes, *Il-4ra* and *Il-6ra*, that are located on autosomes could be detected in *Ftx*^−/−^ immune cells, which confirms a specific effect on X-linked receptor genes (fig. S3A). TLR7, TLR8, and TLR13 trigger, upon activation, the nuclear factor κB pathway (*36*), but neither *Irak1* nor *Ikbkg* (NEMO), two X-linked core effectors of the TLR-signaling pathway (*36*), showed significant changes in their expression level in *Ftx*^−/−^ immune cells (fig. S3B). This suggests either that they are not sensitive to *Xist* perturbation or that their expression is controlled by additional regulation.

### Female mice with impaired XCI develop a splenomegaly during aging

**Fig. 3.**
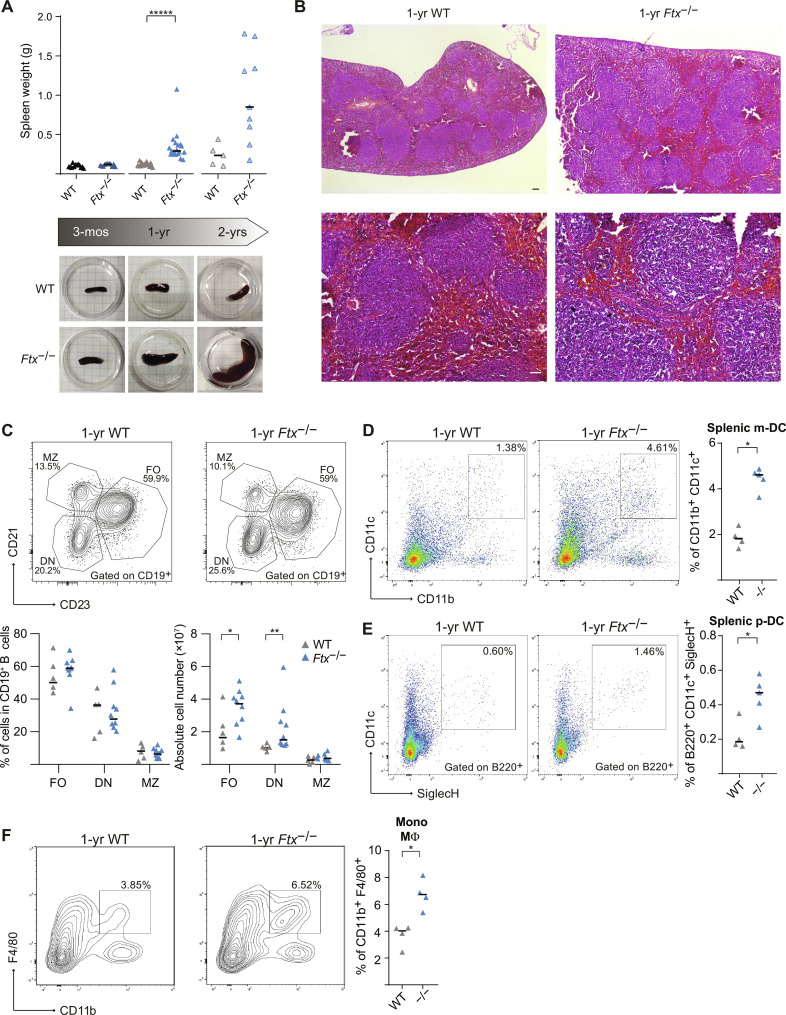
*Ftx*^−/−^ females develop a splenomegaly associated with a deregulation of B and myeloid cell populations. (**A**) Spleen weight of WT and *Ftx*^−/−^ females at 3 months, 1 year, and 2 years of age. Median values are shown (*t* test, *****P < 0.001). Underneath, representative images of WT and *Ftx*^−/−^ spleens from 3-month-, 1-year-, and 2-year-old females. (**B**) Representative images of hematoxylin-eosin staining on sections of spleens from 1-year-old WT and *Ftx*^−/−^ females. Scale bars, 100 μm. (**C**) Representative flow cytometry analysis of follicular (FO; CD21^+^CD23^+^), double-negative (DN; CD21^−^CD23^−^), and marginal zone (MZ; CD21^+^CD23^−^) B cells among CD19^+^ B cells in spleen from 1- to 1.5-year-old WT and *Ftx*^−/−^ females. Percentages and absolute number are shown on the graphs. Median values are shown (Mann-Whitney test, *P < 0.05 and **P < 0.01). (**D**) Representative flow cytometry analysis of splenic myeloid DCs (m-DC) in WT and *Ftx*^−/−^ 1-year-old females. On the right, percentages of CD11b^+^CD11c^+^ splenic m-DC in leucocytes. Each triangle represents a mouse. Median values are shown (Mann-Whitney test, *P < 0.05). (**E**) Same as (D) for CD11c^+^B220^+^SiglecH^+^ splenic plasmacytoid DCs (p-DC) (Mann-Whitney test, *P < 0.05). (**F**) Same as (D) for CD11b^+^F4/80^+^ monocyte/macrophages (Mann-Whitney test, *P < 0.05).

We then characterized changes in the immune system upon XCI perturbation. We could not detect any difference in spleen weight or morphology between *Ftx*^−/−^ and WT females at 3 months of age. In contrast, spleens from 1-year-old and, more markedly, from 2-year-old females appeared significantly larger in *Ftx*^−/−^ conditions (Fig. 3A). Histological analyses did not reveal any defects in cell organization and any signs of fibrosis or of cell infiltration in *Ftx*^−/−^ spleens compared to WT at any age (Fig. 3B and fig. S4A). Both the time-course progression of the splenomegaly and the fact that it never occurred in males (fig. S4B) are consistent with the notion that XCI molecular alteration is the driving force responsible for the phenotype of *Ftx*^−/−^ mice.

The splenomegaly in 1-year-old *Ftx*^−/−^ females resulted from a multilineage cell expansion preferentially targeting myeloid and DCs (table S3). Follicular and CD21^−^CD23^−^ double-negative B cells appeared increased in absolute number but not in percentages, while marginal zone B cell counts remained unchanged upon *Ftx* deficiency (Fig. 3C). In contrast, percentages of both myeloid DCs (Fig. 3D) and plasmacytoid DCs (Fig. 3E) and of Mo/MΦ were significantly higher in *Ftx*^−/−^ compared to WT animals (Fig. 3F). No differences were observed between aged-matched *Ftx*^−/−^ and WT males (table S4).

### Perturbation of XCI leads to autoimmune manifestations

**Fig. 4.**
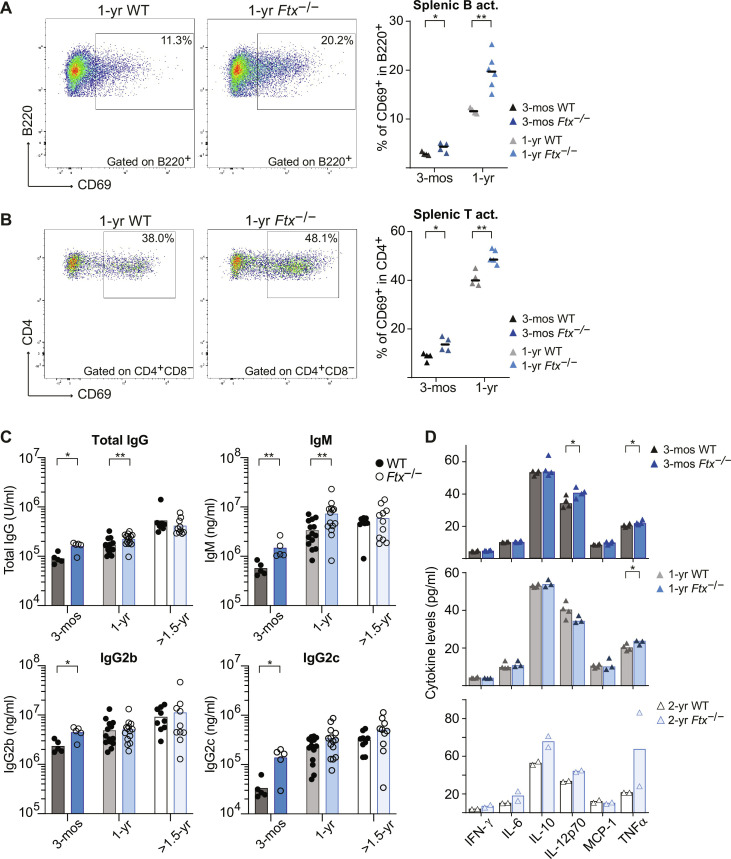
*Ftx* deficiency in female mice promotes signs of inflammation. (**A**) Representative flow cytometry analysis of spontaneously activated B220^+^CD69^+^ B cells in spleen from 1-year-old WT and *Ftx*^−/−^ females. Percentages in leucocytes are shown on the graphs beneath. Each triangle represents a mouse. Median values are shown (Mann-Whitney test, *P < 0.05 and **P < 0.01). (**B**) Same as (A) for spontaneously activated CD4^+^CD69^+^ T cells (Mann-Whitney test, *P < 0.05 and **P < 0.01). (**C**) Total IgG, IgM, IgG2b, and IgG2c natural antibody levels in sera of 3-month-, 1-year, and >1.5-year-old WT or *Ftx*^−/−^ females measured by ELISA. Each circle represents a mouse. Mean values are shown (Mann-Whitney test, *P < 0.05 and **P < 0.01). (**D**) Cytokines levels in the blood analyzed with cytometric bead array assays on sera from 3-month-, 1-year-, or 2-year-old WT and *Ftx*^−/−^ females. Each triangle represents a mouse. Median values are shown (*t* test, *P < 0.05).

Mo/MΦ and DC expansion are typical of inflammation reported in mouse models of SLE, including NZB/W F1 and Yaa mice in which *Tlr7* is overexpressed (*15*, *24*). Accordingly, we observed higher frequencies of spontaneously activated CD69^+^ B and T cells in the spleen of *Ftx*^−/−^ females compared to WT from 3 months of age onward (Fig. 4, A and B). This is associated with higher levels of immunoglobulin M (IgM), total IgG, IgG2b, and IgG2c immunoglobulins in the serum of *Ftx*^−/−^ versus WT females (Fig. 4C). These changes in immune regulation were, however, not associated with any marked increase in cytokine levels in the serum of 3-month- or 1-year-old *Ftx*^−/−^ females (Fig. 4D). Only a mild increase of interleukin-12p70 (IL-12p70) and of tumor necrosis factor–α (TNFα) levels, one of the cytokines induced by TLR7 signaling pathway (*35*), was detected at 3 months and/or 1 year of age, suggesting that cytokine levels are efficiently controlled despite inflammation signs.

### XCI alteration induces a lupus-like syndrome in female mice

**Fig. 5.**
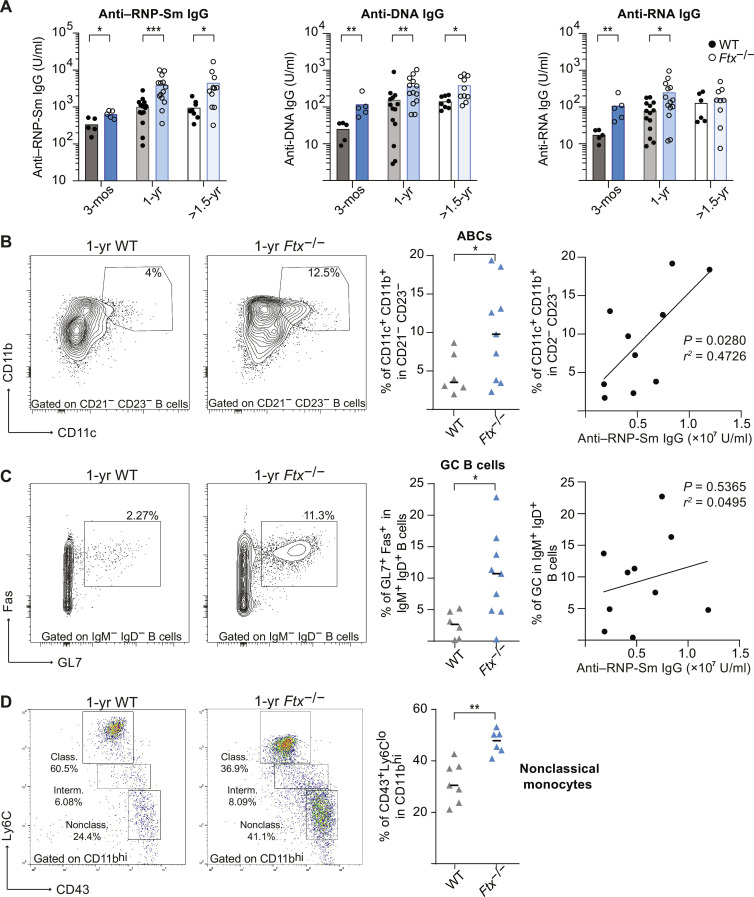
*Ftx* deficiency in female mice induces anti-NA and anti-RNP-Sm autoantibody production and the development of ABC-like cells. (**A**) Anti–RNP-Sm IgG, anti-DNA IgG, and anti-RNA IgG autoantibody levels in sera of 3-month-, 1-year-, and >1.5-year-old WT or *Ftx*^−/−^ females measured by ELISA. Each circle represents a mouse. Mean values are shown (Mann-Whitney test, *P < 0.05, **P < 0.01, and ***P < 0.005). (**B**) Representative flow cytometry analysis of ABC-like cells (CD11c^+^CD11b^+^) among DN (CD21^−^CD23^−^) in spleen from 1-year-old to 1.5-year-old WT and *Ftx*^−/−^ females. Percentages are shown on the graphs. Each triangle represents a mouse. Median values are indicated. (Welch’s *t* test, *P < 0.05). The right panel shows the correlation between the frequency of ABCs (CD11c^+^CD11b^+^) relative to anti–RNP-Sm IgG levels in each *Ftx*^−/−^ female (Pearson correlation). (**C**) Representative flow cytometry analysis of GC cells (Fas^+^GL7^+^) among switch memory B cells (IgM^−^IgD^−^) in spleen from 1-year-old to 1.5-year-old WT and *Ftx*^−/−^ females. Percentages are shown on the graphs. Each triangle represents a mouse. Median values are indicated (Welch’s *t* test, *P < 0.05). The right panel shows the correlation between the frequency of GC cells (Fas^+^GL7^+^) and anti–RNP-Sm IgG Ab levels in each *Ftx*^−/−^ females (Pearson correlation). (**D**) Representative flow cytometry analysis of monocyte populations including nonclassical (CD11b^hi^CD43^+^Ly6C^lo^) scavenger monocytes in the blood of 1-year-old WT and *Ftx*^−/−^ females. Percentages in leucocytes are shown on the graphs beneath. Each triangle represents a mouse. Median values are shown (Mann-Whitney test, **P < 0.01).

Lupus-like syndromes are specifically defined by high quantities of circulating autoantibodies against RNP complex (RNP-Sm) or against NAs including anti-ssRNA and anti-DNA antibodies. Enzyme-linked immunosorbent assay (ELISA) quantifications of anti-RNP-Sm, anti-RNA, and anti-DNA IgG in sera of 3-month-, 1-year-, and >1.5-year-old *Ftx*^−/−^ versus WT females showed significantly higher levels in *Ftx*^−/−^ animals (Fig. 5A). Coincidentally with autoantibodies overproduction, we detected higher frequencies of ABCs and GC B cells in *Ftx*^−/−^ compared to WT spleens (Fig. 5, B and C). In particular, anti-RNP-Sm levels significantly correlated with the percentages of ABCs (Fig. 5B), which are consistently found overrepresented in SLE and other autoimmune disorders (*37*). In contrast, GC B cell percentages did not correlate with anti-RNP-Sm antibody levels (Fig. 5C).

This strongly suggests that ABCs constitute the major producer of this class of autoantibodies as previously reported in human SLE (*38*, *39*). In SLE, ABCs may originate from the extra-follicular pathway and develop into autoreactive plasma cells upon TLR7 signal (*38*). In agreement, the numbers of long-lived plasma cells were increased in the spleen of >1-year-old KO animals (fig. S5, A and B).

Given that the effect of *Xist* perturbation on X-linked gene expression is especially pronounced in Mo/MΦ, we examined the populations of circulating Mo/MΦ in 1-year-old females. We detected an overrepresentation of Ly6C^lo^ nonclassical monocytes in the blood of *Ftx*^−/−^ females (Fig. 5D), a monocyte population recruited at inflammatory tissues in lupus-like contexts (*40*–*42*), but we did not observe signs of inflammation in peripheral tissues like kidneys, a clinical manifestation that is observed at late stages of SLE.

Thus, *Ftx*^−/−^ females progressively develop a splenomegaly during aging, which is accompanied by multiple markers of SLE, including high levels of spontaneous lymphocyte activation, increased percentages of ABC-like and GC B cells, overproduction of immunoglobulins (IgM and IgG), including IgG autoantibodies to NA and RNP-Sm, and a predominance of atypical Ly6C^lo^ monocytes in the circulation.

### XCI impairment triggers an overexpression of target cytokines in TLR7-stimulated macrophages

**Fig. 6.**
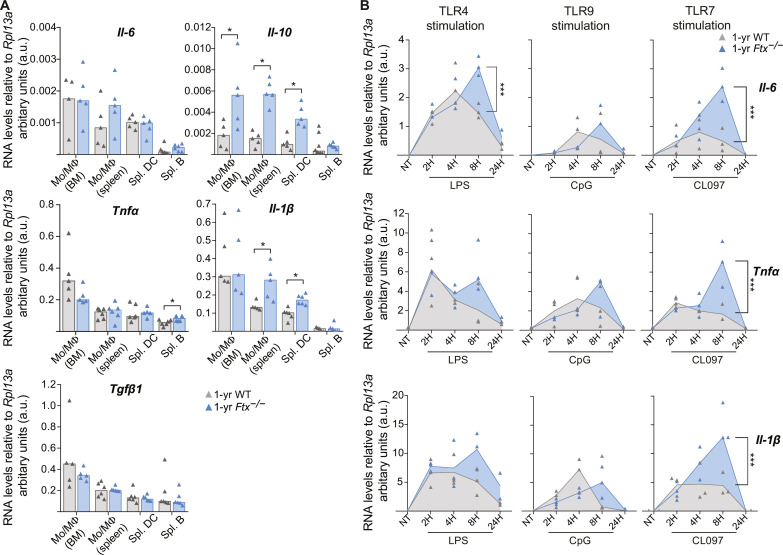
Hyperactive *TLR7* pathway in *Ftx*^−/−^ female macrophages. (**A**) Analysis of cytokine RNA levels by RT-qPCR in the indicated cell population collected from WT or *Ftx*^−/−^ 1-year-old females. Each triangle represents a mouse. Bar plots show median values (*t* test, *P < 0.05). (**B**) RT-qPCR analysis of cytokine RNA levels in BM-derived macrophages (granulocyte-macrophage colony-stimulating factor differentiation of total BM cells) activated with either lipopolysaccharide (LPS; *TLR4* pathway), CpG (*TLR9* pathway), or the *TLR7* agonist CL097. Cells were either not treated (NT) or treated for 2, 4, 8, or 24 hours (2H, 4H, 8H, and 24H, respectively). Each triangle represents a mouse. Median values are shown [analysis of variance (ANOVA), ***P < 0.001].

To test whether TLR7 pathway hyperactivity could contribute to *Ftx*^−/−^ lupus-like phenotype and to identify the targeted cell populations, we first measured the basal expression of cytokine genes normally induced upon activation of TLR7 pathway (i.e., *Tnf*α, *Il-1β*, *Il-6*, and *Il-10*) by RT-qPCR in BM or splenic Mo/MΦ, in splenic DC, and in splenic B cells of 1-year-old females (Fig. 6A).

Basal RNA levels of *Il-10*, *Il-1β*, and *Tnfα* appeared significantly higher in Mo/MΦ and/or in splenic B cells of *Ftx*^−/−^ mice. *Il-6* also displayed similar tendencies. In contrast, *Tgfβ1*, a gene that is not a direct target of the TLR7 pathway, was expressed at the same levels in WT and *Ftx* KO contexts (Fig. 6A). Such changes in the basal production of cytokines may result from secondary effect of the inflammatory/autoimmune phenotype in *Ftx*-deficient mice or from cell-intrinsic mechanisms, and we therefore analyzed the kinetics of TLR-signaling in ex vivo differentiated BM-derived MΦ (fig. S6). These MΦ were then stimulated with TLR ligands, specific for TLR4 [lipopolysaccharide (LPS)], TLR9 (CpG), or TLR7 (CL097), and mRNA expression kinetics of key pro-inflammatory cytokines TNFα, IL-1β, and IL-6 were measured by RT-qPCR. Although all three cytokine transcripts were up-regulated in response to TLR agonist ligands, a higher and sustained expression of these target cytokines was specifically observed in response to *TLR7* activation in *Ftx*-deficient MΦ and not in WT cells (Fig. 6B). A significantly higher up-regulation of *Il-6* was also detected upon TLR4 stimulation in *Ftx*^−/−^ compared to WT MΦ (Fig. 6B).

We conclude that *Ftx*^−/−^ MΦ intrinsically exhibit a hyperfunctional secretory phenotype, characterized by sustained transcriptional activation of the TLR7/TLR8 signaling pathway upon activation. This is likely to induce an overproduction of target cytokines driving adaptive immunity and the development of lupus-like autoimmunity in *Ftx*^−/−^ females.

## DISCUSSION

With recent worldwide waves of viral infections and increased realization that women are more resistant to these infections than men, understanding the basis of sexual dimorphism in immune system competence has emerged as critical to the design of innovative therapeutic strategies (*43*). In this framework, we show that perturbing XCI, a female-specific epigenetic process that is established early during development, directly affects female immune response in mouse adult life. More specifically, certain X-linked genes with immune functions that naturally show a tendency to escape from XCI are expressed from the inactive X in higher percentages of immune cells upon XCI perturbation, resulting in overall higher levels of mRNAs. This deregulation is associated with the progressive development of autoimmune manifestations indicative of an over-reactive immune system. This suggests that, under normal conditions, a low level of escape from XCI of specific X-linked genes leads to a slightly higher dosage of the corresponding immune factors compared to males, which may endow females with enhanced plasticity in immune responses. This hypothesis is corroborated by former observations that correlate higher expression of some X-linked genes (*Tlr7*, *Cxcr3*, and *Tlr8*) in females compared to males with better protection against viral or parasite infections in both humans and mice (*44*–*46*). Moreover, recent studies have identified loss-of-function mutations of *TLR7* associated with severe forms of COVID-19 in young men, demonstrating the essential role of TLR7 pathway in the protective response against severe acute respiratory syndrome coronavirus 2 (*47*–*49*).

Different X-linked genes appear overexpressed upon XCI alteration depending on immune cell types (i.e., *Cxcr3* in T cells; *Tlr7* in B cells; *Tlr7*, *Cybb*, and *Tlr8* in splenic DCs; and *Tlr7*, *Tlr8*, *Tlr13*, *Tasl*, *Il-2rg*, and *Atp7a* in Mo/MΦ), suggesting that various molecular pathways could be affected in immune cells. The number of affected genes might be underestimated because we only tested a subset of immune-related X-linked genes. The phenotype that we observe probably results from the combination of effects ensuing from distinct reactivation events on the Xi in different cell types and the more global changes in gene expression that these initial events triggered. Additional regulatory loops may also be at play. Some overexpressed X-linked genes such as *Tlr7* and *Tasl* are type I interferon (IFN-I)–stimulated genes (*50*). Hence, overexpression of such genes at the mRNA levels could result from a combination of XCI escape and enhanced IFN-I signaling associated with lupus pathogenesis (*51*). Accordingly, we observe higher numbers of pDCs in Ftx-deficient mice compared to WT. IFN-I produced by pDCs is known to propagate anti-NA antibody response and SLE-like disease and could further activate of X-linked IFN-stimulated gene transcription (*51*).

Several lines of evidence support the conclusion that *Tlr7* reactivation is a major contributor to the lupus-like syndrome of *Ftx*^−/−^ females. First, we observe increased monocytosis characterized by the selective expansion in the blood of the Ly6C^lo^ nonclassical monocyte subset, which have been commonly reported in TLR7-driven lupus mouse models (*40*–*42*). Moreover, in the NZM/NZW lupus model, Ly6C^lo^ monocytes, which spontaneously accumulate with age, express high levels of TLR7 protein, and administration of TLR7 agonist ligands accelerates Ly6C^lo^ monocyte augmentation in the blood and promotes nephritis (*40*). Second, GC B cells accumulate in the spleen of *Ftx*^−/−^ mice. Spontaneous GC formation is well described in lupus mouse models and has been shown to be either strictly dependent on B cell–intrinsic TLR7 expression (*52*) or promoted upon enhanced TLR7-signaling (*16*). Last, the formation of pathogenic CD11c^+^ ABCs is significantly enhanced in *Ftx*^−/−^ females, as observed in many lupus-associated conditions in mice (*16*, *53*, *54*) and humans (*38*, *39*). Anti-RNP-Sm autoantibody production strongly correlates with ABC development in *Ftx*^−/−^ females, suggesting a major role of ABC in B cell systemic autoimmunity. Extrafollicular ABCs and their plasma cell products, rather than GC-derived B cells, have been shown to drive the development of pathogenic B cell subsets in spontaneous TLR7-driven lupus models (*16*, *51*).

What is the molecular mechanism underlying reactivation of specific X-linked genes in *Ftx*^−/−^ females? Intriguingly, we noticed that X-linked genes sensitive to *Xist* perturbation tended either to cluster (*Tlr7/Tlr8* and *Atp7a/Tlr13*) or to be located in gene-poor regions (*Cxcr3* and *Cybb*) (Fig. 2A), suggesting a mechanism that operates regionally over several kilobases rather than an action restricted to promoters. In this regard, silencing of *TLR7* and, more generally, of X-linked genes lacking promoter DNA methylation in human B cells is thought to depend on continuous association with *XIST* RNA and *XIST*-dependent H3K27 deacetylation of distal enhancers and not promoters (*25*, *55*). In female mouse Mo/MΦ, the methylation status of the promoter of genes targeted by reactivation is unknown, but it is conceivable that, in the *Ftx*^−/−^ context, reduced deacetylation of local enhancers following exacerbated *Xist* RNA delocalization from the Xi contributes to enhance reactivation of neighboring genes or leads to a spreading of escape to genes subject to XCI in WT cells. Another intriguing observation is the sustained cytokine gene expression upon TLR7 activation of *Ftx*^−/−^ macrophages. Overexpression of *Tlr7* probably leads to TLR7 accumulation at the membrane of late endosome or lysosomes, resulting in prolonged ligand-receptor contact and continuous production of pro-inflammatory cytokines. Deficiency in components of the SMRC8-WDR4-C9ORF72 complex, a regulator of autophagy and lysosomal function, causes inflammation due to excessive endosomal TLR signaling in MΦ (*56*). Alternatively, impaired re-localization of *Xist* RNA to the Xi upon stimulation, a phenomenon that has recently been reported in activated T cells from the MRL/Lpr lupus model (*57*) and in activated B and T cells of *Ciz1*^−/−^ mice suffering from lymphoproliferative disorder (*58*), may lead to sub-efficient secondary repression of *Tlr7* Xi copy. Last, it is tempting to postulate the existence of an active process that would have been evolutionary selected to maintain low levels of XCI escape of specific X-linked genes because this may both confer a greater resistance to infection to female individuals.

To our knowledge, none of the known *XIST* regulators, including *XIST* itself, has been identified as a SLE susceptible locus in genome-wide association studies. Alterations of XCI as a cause of different autoimmune manifestations have not been reported so far but not thoroughly investigated either. Two independent studies have recently reported that (i) *XIST* levels were elevated in blood leucocytes from women with SLE (*59*) and that (ii) transgenic expression of *Xist* lncRNAs in male mice can promote autoantibodies directed against *Xist* RNP in the context of pristane-induced lupus in a permissive genetic background (SJL/J) (*60*). This result seems in apparent contradiction with observations of the present study in which reduced levels of *Xist* in female immune cells, associated with altered XCI, trigger spontaneous systemic autoimmunity. The study by Dou *et al.* (*60*) indicates that *Xist* RNP itself harbors immunogenic properties upon release from dying male cells that are not naturally expressing *Xist*, whereas, in the present study, altered XCI is constitutive and triggers spontaneous autoimmunity in aged female mice of the non-autoimmune prone background (C57/BL6). These two findings are, however, not mutually exclusive and may reflect the wide variety of causes, manifestations, and onset of autoimmunity. Of note, in the spontaneous model of lupus in genetically predisposed mice, disease can develop in the absence of *Xist*. This has been well established in male mice carrying the Y-linked genomic modifier *Yaa*, in which a portion of the X chromosome has translocated onto the Y chromosome, resulting in a twofold increase in *Tlr7* expression (*14*, *54*). In this model, spontaneous systemic autoimmunity is more severe in males than in females, suggesting that it is the translocated *Yaa* locus rather than *Xist* expression, which is the critical factor controlling spontaneous systemic B cell autoimmunity. Further studies using uniformized genetic backgrounds associated with tailored ways to either increase or reduce *Xist* expression will be required to establish the effect of *Xist* RNP and of XCI alteration on the kinetics and types of autoimmune manifestations that are promoted. Together, it is possible that autoreactivity to *Xist* RNP and escape from XCI may both synergize to promote female-biased autoimmunity in the context of tissue damage (*60*).

Molecular and cellular phenotypes resulting from impaired *Xist* expression progress gradually with age. Although we cannot exclude that *Xist* expression depends on *Ftx* in immune cells specifically, it is more likely that XCI perturbation in *Ftx*^−/−^ females that initiates in the embryo (*33*) is transmitted, as such, to the hematopoietic lineage. This perturbation has, however, few effects in immune cells from 3-month-old females—the most marked phenotypic changes are detected around 1-year of age—and does not really affect life expectancy or animal fitness. Whether this evolutive phenotype relates to changes in heterochromatin features that are known to occur during aging (*61*, *62*) and/or whether it is accelerated by the gradual loss of immune cell differentiation potential (*63*) remains to be determined. In this regard, it is tempting to speculate that deregulation of XCI may also contribute to autoimmune conditions specific to postmenopausal women [rheumatoid arthritis (*64*), some forms of Sjögren’s syndrome (*65*), atherosclerosis or of ischemic heart diseases (*43*, *66*), and inflammaging (*67*)] when estrogen levels are low and cannot account for the sexual dimorphism observed in these diseases. In conclusion, we have established a direct link between XCI maintenance and function of the immune system. This paves the way for exploring further the role of XCI regulators in female-biased unexplained forms of autoimmune conditions and opens up alternative potentials for therapeutic strategies.

## MATERIALS AND METHODS

### Generation of *Ftx*-deficient mice

*Ftx*-deficient mice were generated in the Institut Clinique de la Souris (Illkirch, France). The strategy used to create *Ftx*^−/−^ mice on a C57BL/6N background is depicted in fig. S7. *Ftx*^−/−^ females were generated either by mating *Ftx*^−/Y^ males with *Ftx*^+/−^ females or by mating *Ftx*^−/Y^ males with *Ftx*^−/−^ females. Similar results were obtained on *Ftx*^−/−^ females from either cross. Mice were maintained under specific pathogen–free conditions in the animal facility of the Jacques Monod Institute (Paris, France) and handled following the European Community guidelines (project authorization no. 05353.02 approved by the ethical comity of the French Ministry for Scientific Research). Virgin females with no signs of inflammation from self-inflicted or other injuries have been used exclusively.

### Serological analyses

For anti-DNA and anti-RNA IgG ELISA, 96-well ELISA plates were first coated overnight with poly-l-lysine (Sigma-Aldrich) and then overnight with DNA from calf thymus (Sigma-Aldrich) or yeast RNA (Sigma-Aldrich), respectively. For anti–RNP-Sm IgG ELISA, Nunc Maxisorp plates were coated overnight with RNP-Sm antigen (Native Calf Thymus, Arotec). Plates were blocked with phosphate-buffered saline (PBS) with 1% bovine serum albumin (BSA), and sera were then titrated and compared to a standard serum from a pool of SLE1, SLE2, and SLE3 mice. Anti-DNA, anti-RNA, and anti–RNP-Sm IgG are expressed in arbitrary units per milliliter. For each isotype, 1 U/ml corresponds to the standard serum concentration resulting in 50% of the maximum optical density (OD) read at 405 nm. IgGs were revealed with goat anti-mouse biotinylated IgG (SouthernBiotech) followed by alkaline phosphatase–conjugated streptavidin (Jackson ImmunoResearch Laboratories), and absorbance at 405 to 650 nm was read.

For total IgG, IgM, IgG2b, and IgG2c ELISA, 96-well ELISA plates were coated with goat anti-mouse IgG (1 μg/ml; H+L) (Jackson ImmunoResearch Laboratories) in PBS for 2 hours at 37°C and then overnight at 4°C. Serially diluted sera were applied. Specific antibodies were detected with biotinylated goat anti-mouse total IgG, IgM, IgG2b, or IgG2c, respectively (SouthernBiotech), followed by incubation with streptavidin coupled with alkaline phosphatase (Jackson ImmunoResearch Laboratories). Plates were read at 405 to 650 nm with an ELISA reader (Varioskan Flash, Thermo Fisher Scientific). IgM (clone MADNP5), IgG2b (clone MADNP3) from PARIS-anticorps (Cergy-Pontoise, France), and IgG2c (Invitrogen) were used as standards. Results are expressed in nanograms per milliliter. For total IgG, a standard serum from a pool of mice was used. Total IgG levels were expressed in arbitrary units per milliliter (1 U/ml corresponds to the standard serum concentration resulting in 50% of the maximum OD read at 405 nm). Levels of inflammatory cytokines in sera were measured using a cytometric bead array mouse inflammation kit (552364, BD Biosciences) according to the manufacturer’s instructions.

### Flow cytometry analyses

BM, spleen, blood, and peritoneal cavity cells were stained using the following antibodies: CD3 PerCP-Vio770 (130-119-656, Miltenyi Biotec), CD4-allophycocyanin (APC) (130-123-207, Miltenyi Biotec), CD5-APC-Vio770 (130-120-165, Miltenyi Biotec), CD8-fluorescein isothiocyanate (FITC) (130-118-468, Miltenyi Biotec), CD11b APC (553312, BD Pharmingen), CD11c phycoerythrin (PE)–Vio770 (130-110-840, Miltenyi Biotec), CD19-FITC (557398, BD Pharmingen), CD21-APC-Vio770 (130-111-733, Miltenyi Biotec), CD23-PE-Vio770 (130-118-764, Miltenyi Biotec), CD38-PE (130-123-571, Miltenyi Biotec), CD43-PE (130-112-887, Miltenyi Biotec), CD69-PE (130-115-575, Miltenyi Biotec), CD138 PE-Vio615 (130-108-989, Miltenyi Biotec), F4/80 FITC (130-117-509, Miltenyi Biotec), Ter119 PE (130-112-909, Miltenyi Biotec), SiglecH APC-Vio770 (130-112-299, Miltenyi Biotec), B220-APC (130-110-847, Miltenyi Biotec), B220 VioBlue (130-110-851, Miltenyi Biotec), IgM-VioBlue (130-116-318, Miltenyi Biotec), IgD-PE (130-111-496, Miltenyi Biotec), GL7-PE-Cy7 (144619, BioLegend), Ly6C-FITC (130111-915, Miltenyi Biotec), streptavidin FITC (554060, BD Biosciences), CD138 BV605 (563147, BD-Horizon), CD23 BV605 (101637, BioLegend), I-A/I-E BV711 (107643, BioLegend), CD19 BV786 (563333, BD Horizon), T and B cell activation antigen (GL7) PE (561530, BD Pharmingen), CD95 PE-Cy7 (557653, BD Pharmingen), IgM APC-eFluor 780 (47-5790-82, Invitrogen), CD45R/B220 APC/cyanine7 (103224, BioLegend), CD11b eFluor 450 (48-0112-82, eBioscience), CD267 (TACI) BV421 (742840, BD Biosciences), IgD BUV395 (564274, BD Horizon), streptavidin APC (4317-82, eBioscience), CD3 Biotin (100304, BioLegend), Biotin CD11c (568970, BD Biosciences), CD21 PercP Cy5.5 (562797, BD Biosciences), and Fixable Viability Dye eFluor 506 (65-0866-18, Invitrogen) following the recommendations of the manufacturers. Flow cytometry was conducted on a BD FACSAria Fusion (BD Biosciences) at the ImagoSeine platform of the Jacques Monod Institute (Paris, France). The instrument is equipped with 18 detectors and five lasers (ultraviolet, 355 nm, 20 mW; violet, 405 nm, 10 mW; blue, 488 nm, 13 mW; yellow-green, 561 nm, 50 mW; and red, 633 nm, 11 mW). The Cytometer Setup and Tracking RUO beads (lot ID 82169) were used to establish reference fluorescence intensities for measuring instrument sensitivity over time. At least 50,000 single cells were acquired. Data files were exported to Flow Cytometry Standard (FCS) file 3.1. All manual analysis was performed using FlowJo 10.8.1 (BD Biosciences). Signal stability over time was checked using flowClean plugin. Forward scatter (FSC) height and area are plotted against each other and used to eliminate doublets and aggregates.

### Cell sorting and activation

#### Magnetic sorting

After isolation, spleen or BM cells were stained for 30 min on ice with Anti-CD23 Magnetic Microbeads (130-098-784, Miltenyi Biotec), Anti-F4/80 Magnetic Microbeads (130-110-443, Miltenyi Biotec), Anti-CD11b Magnetic Microbeads (130-126-725, Miltenyi Biotec), CD11c Magnetic Microbeads (130-108-338, Miltenyi Biotec), Anti-CD90.2 Magnetic Microbeads (130-121-278, Miltenyi Biotec), or Anti-CD19 Magnetic Microbeads (130-121-301, Miltenyi Biotec); washed with 1× PBS with 3% fetal bovine serum (FBS); and centrifuged at 300*g* for 10 min. Cells suspension was purified on LS column (130–042-401, Miltenyi Biotec). Purity of sorted cells was controlled by cytometry using corresponding antibodies.

For differentiation of BM-derived macrophages (BMDMs), total BM cells were cultured with granulocyte-macrophage colony-stimulating factor (100 ng/ml; 130-095-742, Miltenyi Biotec) in complete RPMI 1640 (RPMI 1640 and GlutaMAX, 10% FBS, 1 mM sodium pyurvate, 10 mM Hepes, 0.1 mM nonessential amino acids (NEAA), and 50 μM β-mercaptoethanol). Differentiation efficiency was checked by cytometry using a CD11c, CD11b, F4/80, CD3, and B220 antibody panel.

#### Cell activation

BMDMs were stimulated with LPS (1 ng/ml; Sigma-Aldrich) for TLR4 activation, CpG ODN 2395 (100 nM; tlrl-2395, InvivoGen) for TLR9 activation, or CL097 (50 nM; tlrl-c97, InvivoGen) for TLR 7 activation. Matched negative controls ODN 5328 (InvivoGen) or CL075 (InvivoGen) were used for ODN 2395 and CL097, respectively.

### RNA fluorescence in situ hybridization

#### Cell preparation

Magnetic sorted cells or fluorescence-activated cell sorting–sorted cells were allowed to sediment on poly-lysine–coated slides (Thermo Fisher Scientific) in a 50-μl drop of 1× PBS for 15 min at room temperature, fixed for 10 min in an ice-cold 3% paraformaldehyde/1× PBS solution (Electron Microscopy Sciences), and permeabilized for 5 to 10 min in ice-cold cytoskeletal (CSK) buffer [10 mM 30 Pipes, 300 mM sucrose, 100 mM NaCl, and 3 mM MgCl_2_ (pH6.8)] supplemented with 0.5% Triton X-100 (Sigma-Aldrich) and 2 mM vanadyl-ribonucleoside complex (VRC; New England Biolabs).

#### Probe preparation

One microgram of purified fosmid/bacterial artificial chromosome DNA purified using standard alkaline lysis protocol was labeled with fluorescent deoxyuridine triphosphates (SpectrumOrange and SpectrumGreen from Abott Molecular and Cy5-UTPs from GE HealthCare Life Sciences) in a 50-μl nick-translation reaction for 3 hours at 15°C. *Xist*, p510 plasmid (*68*); *Ftx*, fosmid probe (WI1-1177B13, BACPAC); *Tlr7*, fosmid probes (WI1-1548 K9, WI1-977A7, BACPAC); and *Tasl*, fosmid probes (WI1-1708P12, WI1-841O8, BACPAC).

#### Hybridization

One hundred nanograms of probe was co-precipitated with 3 μg of *Cot-I* DNA (Invitrogen) and 10 μg of Sheared Salmon Sperm DNA (Invitrogen) using 1/10 3 M NaOAc and 2.5 volumes of ethanol for 3 hours at −20°C. Precipitated probes were washed with 70% ethanol and resuspended in formamide (deionized formamide > 99.5%; Sigma-Aldrich) and then denatured for 7 min at 75°C. After probe denaturation, an equivalent volume of 2× hybridization buffer [4× saline sodium citrate (SSC; Ambion), 20% dextran sulfate, BSA (2 mg/ml; New England Biolabs), and 2 mM VRC (New England Biolabs)] was added, and slides were hybridized overnight at 37°C in a humid chamber. The slides were subsequently washed three times in 50% formamide/2× SSC (pH 7.2) at 42°C for 5 min each and three times in 2× SSC at 42°C for 5 min each. Slides were mounted in Vectashield containing 4′,6-diamidino-2-phenylindole (DAPI) (Vector Laboratories).

### Microscopy and image analysis

Images were taken on an Axioplan 2 Imaging fluorescence microscope (Zeiss) with a cooled Coolsnap camera (Roper Scientifics) or a DMI-6000 inverted fluorescence microscope with a motorized stage (Leica) and a charge-coupled device camera HQ2 (Roper Scientific), both controlled by the MetaMorph 7.04 software (Roper Scientifics), using a Plan-NEOFLUAR 63×/1.25 oil objective (Zeiss), a Plan-NEOFLUAR 100×/1.30 oil objective (Zeiss), or a HCX PL APO 63×/1.4 oil objective (Leica). Optical sections were collected at 0.2-mm steps through each nucleus at different wavelengths (in nanometers) {Zeiss: DAPI (345, 455), FITC (488, 507), and CY3 (625, 660); Leica: DAPI (360, 470), FITC (470, 525), and CY3 (550, 570)}. Approximately 40 optical sections per nucleus were collected. Stacks were processed using Icy (http://icy.bioimageanalysis.org), and the images are represented as two-dimensional projections of the stacks (maximum projection).

## Supplementary Materials

### This PDF file includes:

Figs. S1 to S7 References
