# Supplementary material for: Altered X-chromosome inactivation predisposes to autoimmunity
Source: Sci Adv. 2024 May 3;10(18):eadn6537. doi: 10.1126/sciadv.adn6537 (PMC11068014; doi:10.1126/sciadv.adn6537)
Supplement: Supplementary file 1 — Figs. S1 to S7 References [file sciadv.adn6537_sm.pdf]

Science Advances
AAAS

Supplementary Materials for

Altered X-chromosome inactivation predisposes to autoimmunity

Christophe Huret *et al.*

Corresponding author: Claire Rougeulle, claire.rougeulle@u-paris.fr; Céline Morey, celine.morey@inserm.fr

Sci. Adv. **10**, eadn6537 (2024)
DOI: 10.1126/sciadv.adn6537

The PDF file includes:

Figs. S1 to S7
References

Other Supplementary Material for this manuscript includes the following:

Tables S1 to S5

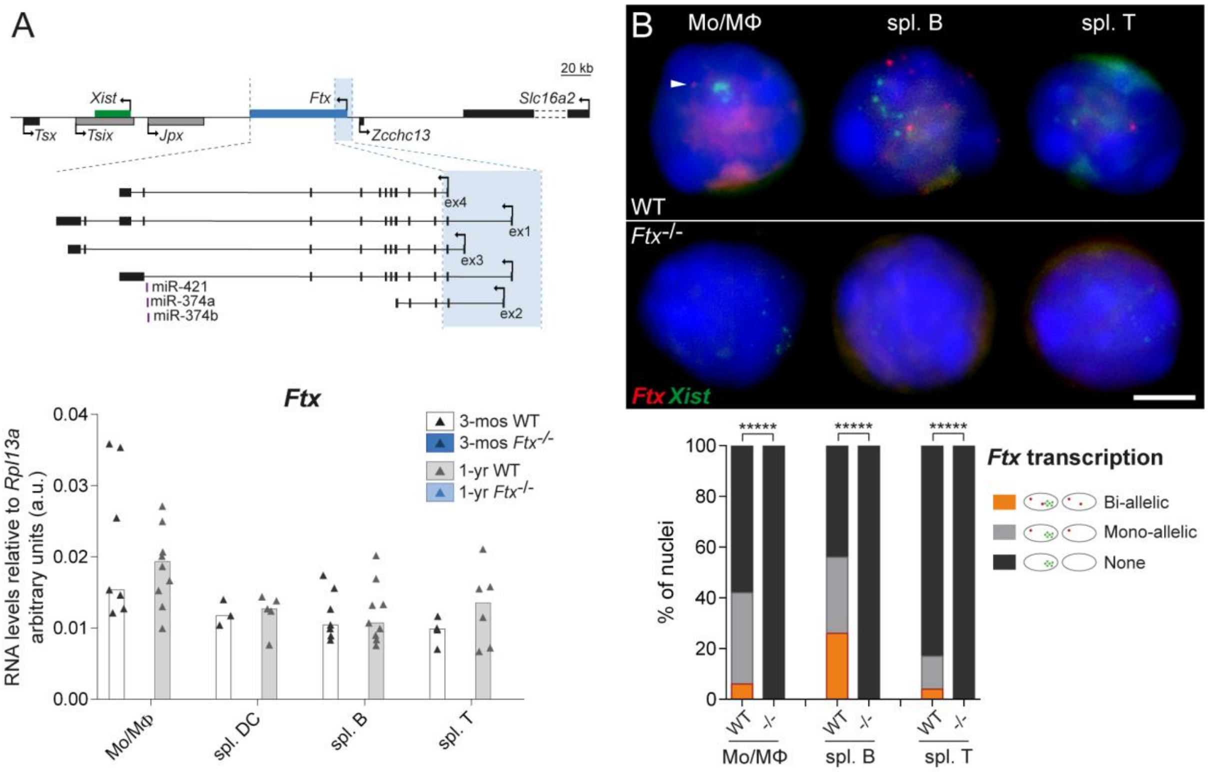

Figure S1. Lack of *Ftx* transcripts in *Ftx*$^{-/-}$ immune cells.

(**A**) Map of the mouse X-inactivation center showing the boundaries of the *Ftx* promoter deletion and, underneath, the effect on the various known isoforms of *Ftx* transcripts. Underneath, RT-qPCR analysis of *Ftx* RNA levels in indicated cell populations collected either from 3-month- or from 1-year-old WT or *Ftx*$^{-/-}$ female mice. qPCR yielded no PCR products in KO animals. Each triangle represents a mouse. Bar plots show median values. (**B**) Double RNA-FISH for *Ftx* (red) and for *Xist* (green) in indicated cell populations collected from 1-year-old WT or *Ftx*$^{-/-}$ females. Note that *Xist* lncRNAs tend to be delocalized from the Xi even in WT mice as previously described (*21*). (χ^2 *test*; *****p-*values* < 0.001; $N \geq 2$ mice; $n \geq 100$ nuclei/mice).

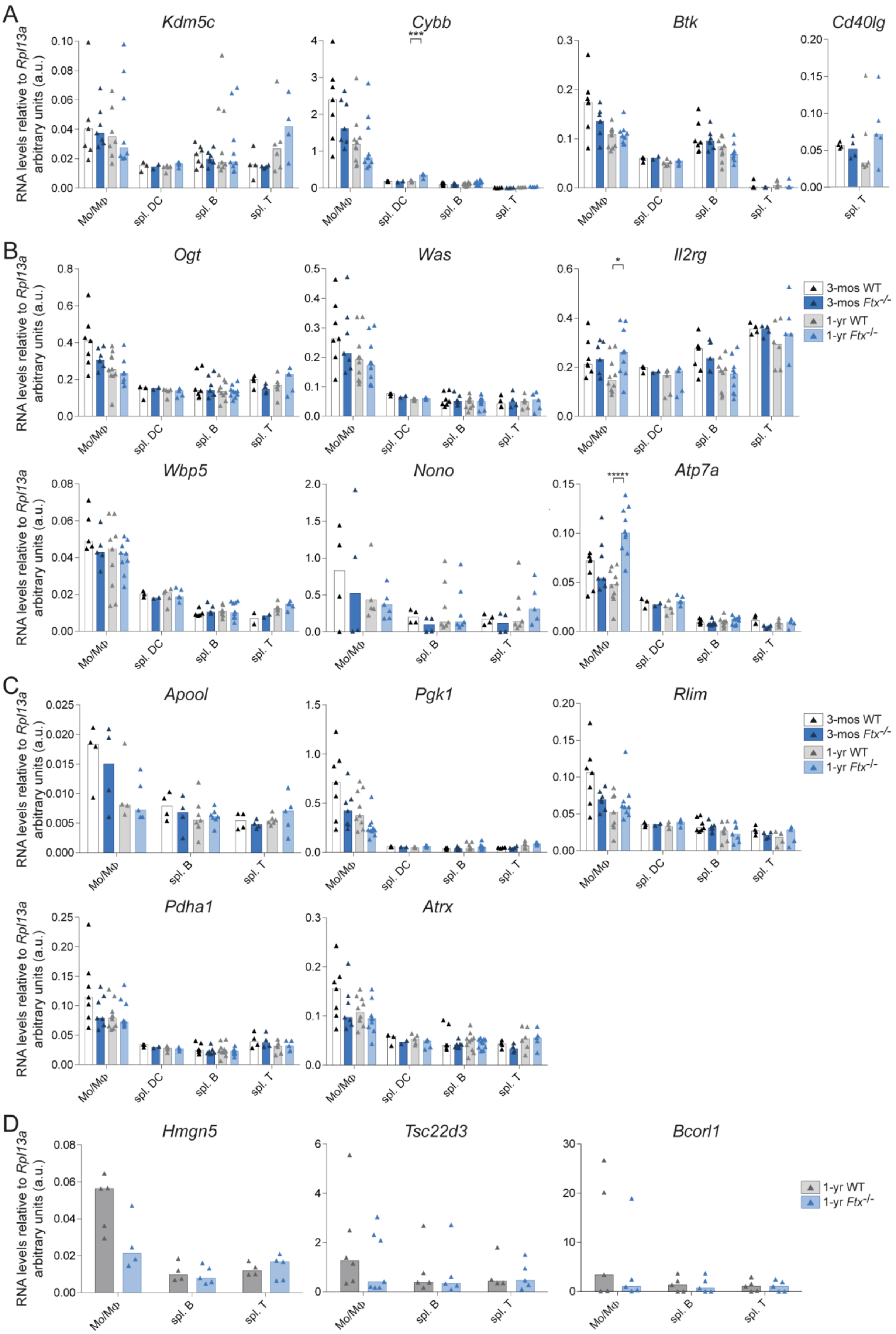

A
Kdm5c
Cybb
Btk
Cd40lg
B
Ogt
Was
Il2rg
Wbp5
Nono
Atp7a
C
Apool
Pgk1
Rlim
Pdha1
Atrx
D
Hmgn5
Tsc22d3
Bcorl1
RNA levels relative to Rpl13a arbitrary units (a.u.)
Mo/Mφ
spl. DC
spl. B
spl. T
3-mos WT
3-mos Ftx-/-
1-yr WT
1-yr Ftx-/-

Figure S2. Expression levels of individual X-linked genes.

(**A**) Expression of genes known to escape from XCI analysed by RT-qPCR in the indicated cell populations collected either from 3-month- or from 1-year-old WT or *Ftx*$^{-/-}$ females. Each triangle represents a mouse. Bar plots show median values. (*t-test*; ****p-values* < 0.005). (**B**) Same as in panel (**A**) for genes with immune functions. (*t-test*; **p-values* < 0.05; ******p-values* < 0.001). (**C**) Same as in panel (**A**) for housekeeping genes. (**D**) Same as in panel (**A**) for genes showing a tendency to be expressed at a lower level in *Ftx*$^{-/-}$ compared to WT immune cells.

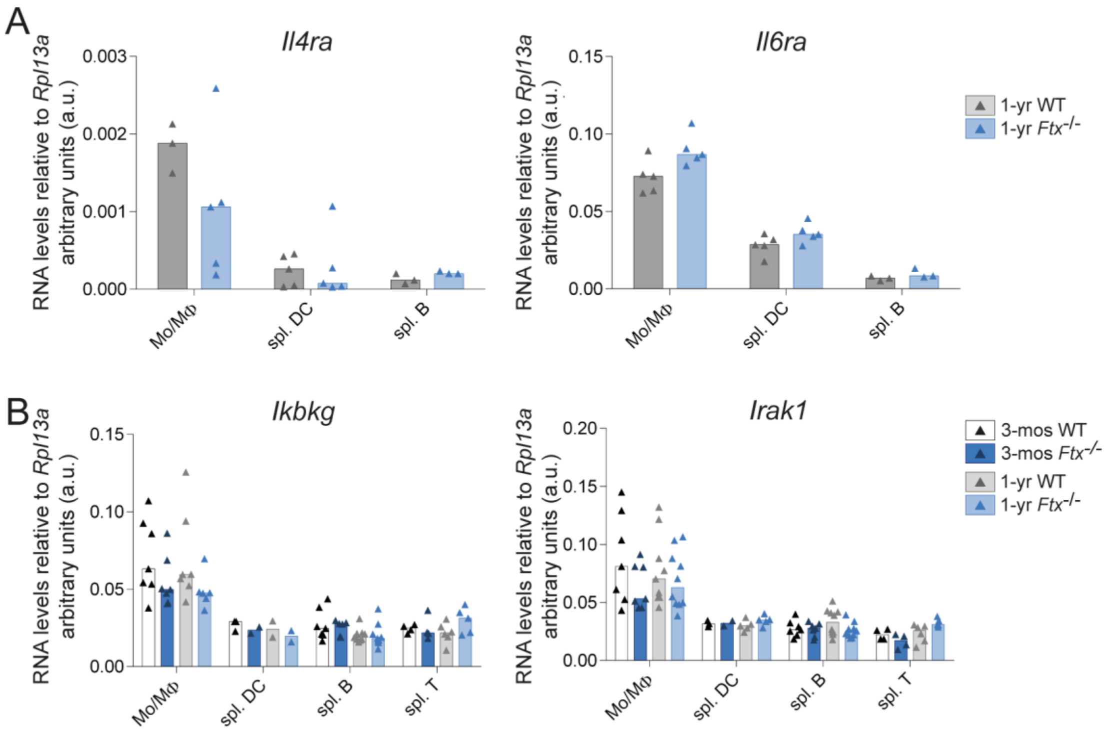

Figure S3. Expression levels of autosome-linked *IL4* and *IL6* membrane receptors and of X-linked members of the NF-κB signalling pathway.

(**A**) Expression of autosomal genes encoding cytokine membrane receptors analysed by RT-qPCR in the indicated cell populations collected either from 3-month- or from 1-year-old WT or *Ftx*$^{-/-}$ females. Each triangle represents a mouse. Bar plots show median values. (**B**) Same as in panel (**A**) for X-linked member of the NF-κB pathway, *Ikbkg* (NEMO) and *Irak1*.

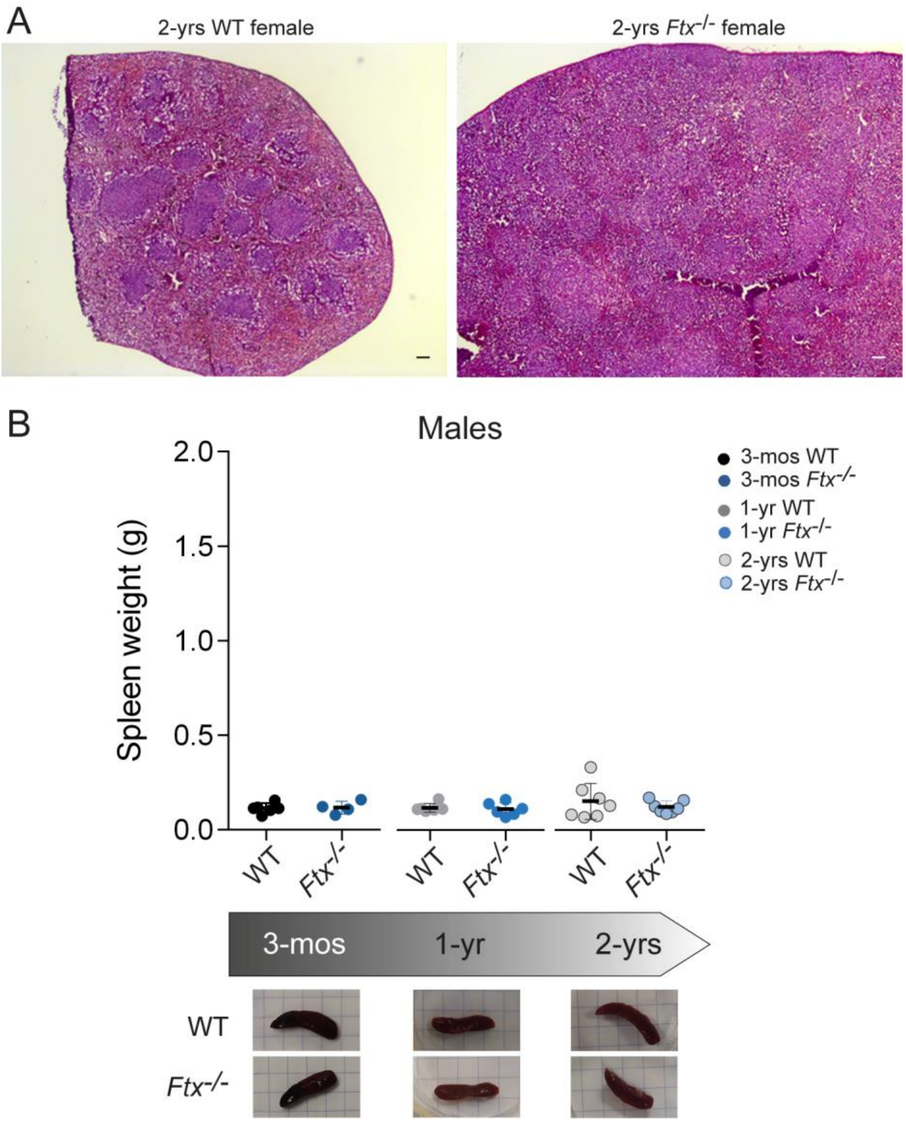

Figure S4. Morphological and histological analyses of spleens from 2-year-old female and male mice.

(**A**) Hematoxylin-Eosin staining of spleen sections from 2-year-old WT or *Ftx*$^{-/-}$ females. (**B**) Spleen weight of wild-type (WT) and *Ftx* KO males at 3-months, 1-year and 2-years of age. Each triangle represents a mouse. Median values are shown. (*t-test*, not significant). Underneath, representative images of WT and *Ftx*$^{-/-}$ spleens from 3-month-, 1-year- and 2-year-old males.

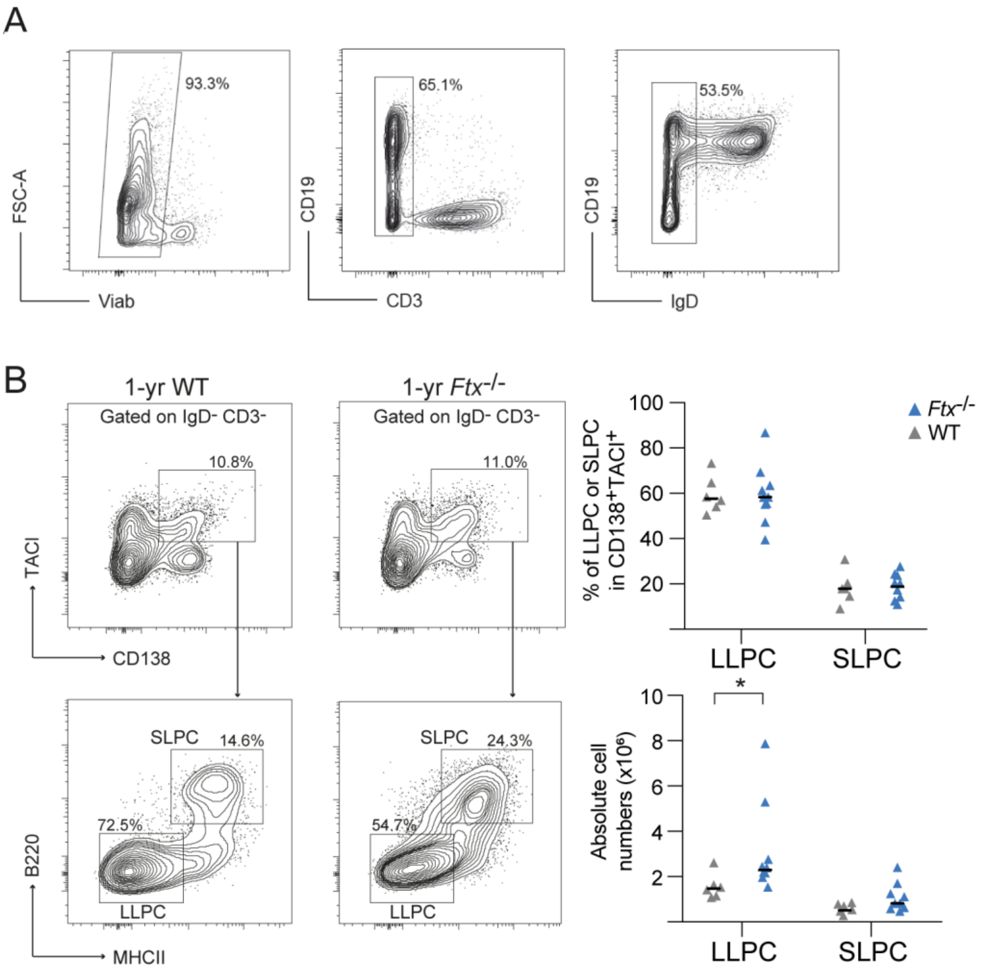

Figure S5. ***Ftx*$^{-/-}$ females display high numbers of long-lived plasma cells in the spleen.** (**A**) Spleen cells from 1-1.5-year-old WT and *Ftx*$^{-/-}$ females were isolated and stained for flow cytometry analysis. Cells were pre-gated on FSC-A and SSC-A then doublets were excluded by gating on FSC-W and SSC-W and live cells were selected by gating on Via dye negative cells. Gating on IgD^{-} and CD3^{-} cells enriched for cells that include TACI^{+} CD138^{+} plasma cells. (**B**) Representative flow cytometry analysis of long-lived plasma cells (LLPC, B220^{-}MHC2^{-}) and short-lived plasma cells (SLPC, B220^{+}MHC2^{+}) among CD3^{-} IgD^{-} TACI^{+} CD138^{+} cells in spleen from 1-1.5-year-old WT and *Ftx*$^{-/-}$ females. Percentages and absolute number from individual mice are shown. Median values. (*Mann-Whitney test*, *p-values < 0.05).

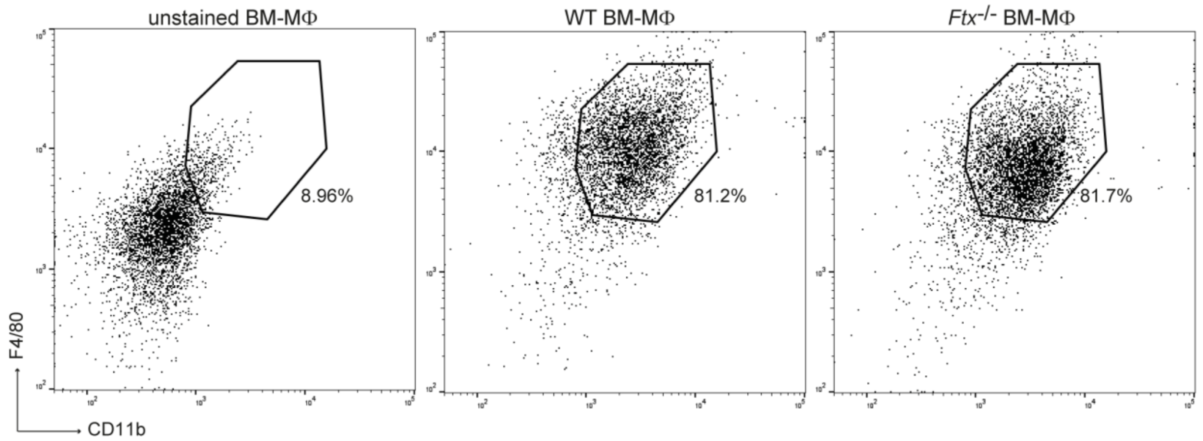

Figure S6. Flow cytometry profiles of BM-derived macrophages.

BM-derived cells from WT or *Ftx*$^{-/-}$ mice differentiated into macrophages upon GM-CSF treatment were stained with F4/80 and CD11b antibodies. The vast majority of cells expressed both markers as compared to the unstained control indicating efficient conversion into macrophages.

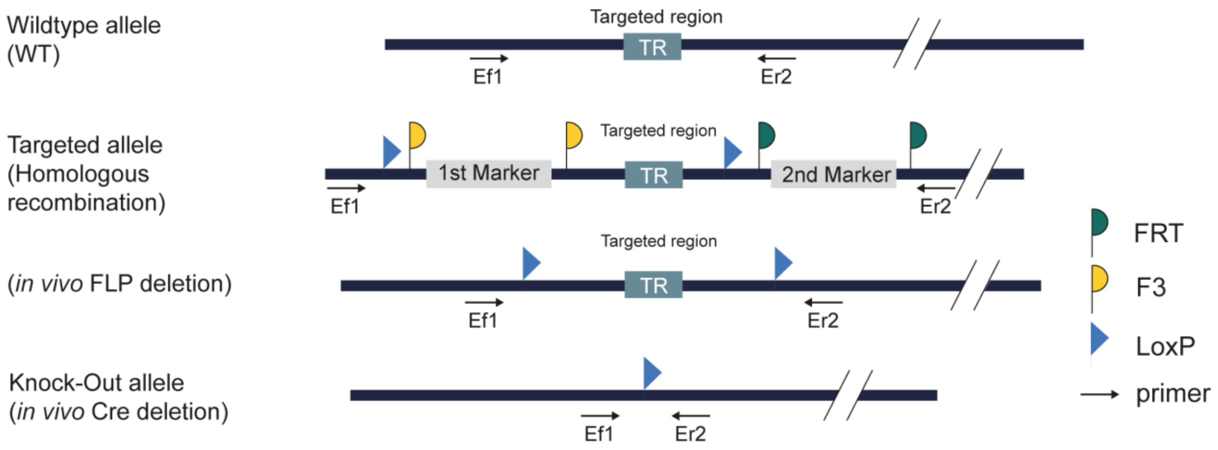

Figure S7. Strategy used to generate the *Ftx* deficient mouse line.

Independent targeted C57BL/6N ES cell lines, were injected into C57BL/6N blastocysts to create germline chimeras. The targeted allele derived from recombinant ES cells was transmitted to female offspring from chimeric males. Mice carrying *Ftx* promoter deletion were obtained after two-rounds of *in vivo* recombination with FLP and Cre driver mice successively resulting in a single *LoxP* site left at the position of the deleted region. Ef1 and Er2 primer pair was used for PCR genotyping.

REFERENCES AND NOTES

1. H. Schurz, M. Salie, G. Tromp, E. G. Hoal, C. J. Kinnear, M. Möller, The X chromosome and sex-specific effects in infectious disease susceptibility. *Hum. Genomics* **13**, 2 (2019).

2. R. Sauteraud, J. M. Stahl, J. James, M. Englebright, F. Chen, X. Zhan, L. Carrel, D. J. Liu, Inferring genes that escape X-Chromosome inactivation reveals important contribution of variable escape genes to sex-biased diseases. *Genome Res.* **31**, 1629–1637 (2021).

3. I. Sierra, M. C. Anguera, Enjoy the silence: X-chromosome inactivation diversity in somatic cells. *Curr. Opin. Genet. Dev.* **55**, 26–31 (2019).

4. S. C. Credendino, C. Neumayer, I. Cantone, Genetics and epigenetics of sex bias: Insights from human cancer and autoimmunity. *Trends Genet.* **36**, 650–663 (2020).

5. G. J. Yuen, Autoimmunity in women: An eXamination of eXisting models. *Clin. Immunol.* **210**, 108270 (2020).

6. S. L. Klein, K. L. Flanagan, Sex differences in immune responses. *Nat. Rev. Immunol.* **16**, 626–638 (2016).

7. R. H. Scofield, G. R. Bruner, B. Namjou, R. P. Kimberly, R. Ramsey-Goldman, M. Petri, J. D. Reveille, G. S. Alarcón, L. M. Vilá, J. Reid, B. Harris, S. Li, J. A. Kelly, J. B. Harley, Klinefelter's syndrome (47,XXY) in male systemic lupus erythematosus patients: Support for the notion of a gene-dose effect from the X chromosome. *Arthritis Rheum.* **58**, 2511–2517 (2008).

8. V. M. Harris, R. Sharma, J. Cavett, B. T. Kurien, K. Liu, K. A. Koelsch, A. Rasmussen, L. Radfar, D. Lewis, D. U. Stone, C. E. Kaufman, S. Li, B. Segal, D. J. Wallace, M. H. Weisman, S. Venuturupalli, J. A. Kelly, M. E. Alarcon-Riquelme, B. Pons-Estel, R. Jonsson, X. Lu, J.-E. Gottenberg, J.-M. Anaya, D. S. Cunninghame-Graham, A. J. W. Huang, M. T. Brennan, P. Hughes, I. Alevizos, C. Miceli-Richard, E. C. Keystone, V. P. Bykerk, G. Hirschfield, G. Xie, K. A. Siminovitch, W.-F. Ng, G. Nordmark, S. M. Bucher, P. Eriksson, R. Omdal, N. L. Rhodus, M. Rischmueller, M. Rohrer, M. Wahren-Herlenius, T. Witte, X. Mariette, C. J. Lessard, J. B. Harley, K. L. Sivils, R. H. Scofield, Klinefelter's syndrome (47,XXY) is in excess among men with Sjögren's syndrome. *Clin. Immunol.* **168**, 25–29 (2016).

9. R. H. Scofield, V. M. Lewis, J. Cavitt, B. T. Kurien, S. Assassi, J. Martin, O. Gorlova, P. Gregersen, A. Lee, L. G. Rider, T. O'Hanlon, S. Rothwell, J. Lilleker, Myositis Genetics Consortium, Xiaoxi Liu, Y. Kochi, C. Terao, A. Igoe, W. Stevens, J. Sahhar, J. Roddy, M. Rischmueller, S. Lester, S. Proudman, S. Chen, M. A. Brown, M. D. Mayes, J. A. Lamb, F. W. Miller, 47XXY and 47XXX in Scleroderma and Myositis *ACR Open Rheumatol.* **4**, 528–533 (2022).

10. M. Cattalini, M. Soliani, M. C. Caparello, R. Cimaz, Sex differences in pediatric rheumatology. *Clin. Rev. Allergy Immunol.* **56**, 293–307 (2019).

11. M. Souyris, J. E. Mejía, J. Chaumeil, J.-C. Guéry, Female predisposition to TLR7-driven autoimmunity: Gene dosage and the escape from X chromosome inactivation. *Semin. Immunopathol.* **41**, 153–164 (2019).

12. C. Bost, M. I. Arleevskaya, W. H. Brooks, S. Plaza, J.-C. Guery, Y. Renaudineau, Long non-coding RNA Xist contribution in systemic lupus erythematosus and rheumatoid arthritis. *Clin. Immunol.* **236**, 108937 (2022).

13. A. Youness, C.-H. Miquel, J.-C. Guéry, Escape from X chromosome inactivation and the female predominance in autoimmune diseases. *Int. J. Mol. Sci.* **22**, 1114 (2021).

14. P. Pisitkun, J. A. Deane, M. J. Difilippantonio, T. Tarasenko, A. B. Satterthwaite, S. Bolland, Autoreactive B cell responses to RNA-related antigens due to TLR7 gene duplication. *Science* **312**, 1669–1672 (2006).

15. J. A. Deane, P. Pisitkun, R. S. Barrett, L. Feigenbaum, T. Town, J. M. Ward, R. A. Flavell, S. Bolland, Control of toll-like receptor 7 expression is essential to restrict autoimmunity and dendritic cell proliferation. *Immunity* **27**, 801–810 (2007).

16. G. J. Brown, P. F. Cañete, H. Wang, A. Medhavy, J. Bones, J. A. Roco, Y. He, Y. Qin, J. Cappello, J. I. Ellyard, K. Bassett, Q. Shen, G. Burgio, Y. Zhang, C. Turnbull, X. Meng, P. Wu, E. Cho, L. A. Miosge, T. D. Andrews, M. A. Field, D. Tvorogov, A. F. Lopez, J. J. Babon, C. A. López, Á. Gónzalez-Murillo, D. C. Garulo, V. Pascual, T. Levy, E. J. Mallack, D. G. Calame, T. Lotze, J. R. Lupski, H. Ding, T. R. Ullah, G. D. Walters, M. E. Koina, M. C. Cook, N. Shen, C. de Lucas Collantes, B. Corry, M. P.

Gantier, V. Athanasopoulos, C. G. TLR7 gain-of-function genetic variation causes human lupus. *Nature* **605**, 349–356 (2022).

17. A. Loda, S. Collombet, E. Heard, Gene regulation in time and space during X-chromosome inactivation. *Nat. Rev. Mol. Cell Biol.* **23**, 231–249 (2022).

18. M. Souyris, C. Cenac, P. Azar, D. Daviaud, A. Canivet, S. Grunenwald, C. Pienkowski, J. Chaumeil, J. E. Mejía, J.-C. Guéry, TLR7 escapes X chromosome inactivation in immune cells. *Sci. Immunol.* **3**, eaap8855 (2018).

19. T. Tukiainen, A.-C. Villani, A. Yen, M. A. Rivas, J. L. Marshall, R. Satija, M. Aguirre, L. Gauthier, M. Fleharty, A. Kirby, B. B. Cummings, S. E. Castel, K. J. Karczewski, F. Aguet, A. Byrnes, GTEx Consortium;, T. Lappalainen, A. Regev, K. G. Ardlie, N. Hacohen, D. G. MacArthur, Landscape of X chromosome inactivation across human tissues. *Nature* **550**, 244–248 (2017).

20. J. B. Berletch, W. Ma, F. Yang, J. Shendure, W. S. Noble, C. M. Disteche, X. Deng, Escape from X inactivation varies in mouse tissues. *PLOS Genet.* **11**, e1005079 (2015).

21. J. Wang, C. M. Syrett, M. C. Kramer, A. Basu, M. L. Atchison, M. C. Anguera, Unusual maintenance of X chromosome inactivation predisposes female lymphocytes for increased expression from the inactive X. *Proc. Natl. Acad. Sci. U.S.A.* **113**, E2029–2038 (2016).

22. C. M. Syrett, V. Sindhava, I. Sierra, A. H. Dubin, M. Atchison, M. C. Anguera, Diversity of epigenetic features of the inactive X-chromosome in NK cells, dendritic cells, and macrophages. *Front. Immunol.* **9**, 3087 (2018).

23. C. M. Syrett, I. Sierra, Z. T. Beethem, A. H. Dubin, M. C. Anguera, Loss of epigenetic modifications on the inactive X chromosome and sex-biased gene expression profiles in B cells from NZB/W F1 mice with lupus-like disease. *J. Autoimmun.* **107**, 102357 (2020).

24. B. J. Helyer, J. B. Howie, Renal disease associated with positive lupus erythematosus tests in a crossbred strain of mice. *Nature* **197**, 197–197 (1963).

25. B. Yu, Y. Qi, R. Li, Q. Shi, A. T. Satpathy, H. Y. Chang, B cell-specific XIST complex enforces X-inactivation and restrains atypical B cells. *Cell* **184**, 1790–1803.e17 (2021).

26. T. Yang, J. Ou, E. Yildirim, Xist exerts gene-specific silencing during XCI maintenance and impacts lineage-specific cell differentiation and proliferation during hematopoiesis. *Nat. Commun.* **13**, 4464 (2022).

27. E. Yildirim, J. E. Kirby, D. E. Brown, F. E. Mercier, R. I. Sadreyev, D. T. Scadden, J. T. Lee, Xist RNA is a potent suppressor of hematologic cancer in mice. *Cell* **152**, 727–742 (2013).

28. L. Yang, J. E. Kirby, H. Sunwoo, J. T. Lee, Female mice lacking Xist RNA show partial dosage compensation and survive to term. *Genes Dev.* **30**, 1747–1760 (2016).

29. L. Yang, E. Yildirim, J. E. Kirby, W. Press, J. T. Lee, Widespread organ tolerance to Xist loss and X reactivation except under chronic stress in the gut. *Proc. Natl. Acad. Sci. U.S.A.* **117**, 4262–4272 (2020).

30. C. Chureau, S. Chantalat, A. Romito, A. Galvani, L. Duret, P. Avner, C. Rougeulle, Ftx is a non-coding RNA which affects Xist expression and chromatin structure within the X-inactivation center region. *Hum. Mol. Genet.* **20**, 705–718 (2011).

31. C. Chureau, M. Prissette, A. Bourdet, V. Barbe, L. Cattolico, L. Jones, A. Eggen, P. Avner, L. Duret, Comparative sequence analysis of the X-inactivation center region in mouse, human, and bovine. *Genome Res.* **12**, 894–908 (2002).

32. G. Furlan, N. Gutierrez Hernandez, C. Huret, R. Galupa, J. G. van Bemmel, A. Romito, E. Heard, C. Morey, C. Rougeulle, The Ftx noncoding locus controls X chromosome inactivation independently of its RNA products. *Mol. Cell* **70**, 462–472.e8 (2018).

33. Y. Hosoi, M. Soma, H. Shiura, T. Sado, H. Hasuwa, K. Abe, T. Kohda, F. Ishino, S. Kobayashi, Female mice lacking Ftx lncRNA exhibit impaired X-chromosome inactivation and a microphthalmia-like phenotype. *Nat. Commun.* **9**, 3829 (2018).

34. M. Soma, Y. Fujihara, M. Okabe, F. Ishino, S. Kobayashi, Ftx is dispensable for imprinted X-chromosome inactivation in preimplantation mouse embryos. *Sci. Rep.* **4**, 5181 (2014).

35. N. Jiwrajka, M. C. Anguera, The X in seX-biased immunity and autoimmune rheumatic disease. *J. Exp. Med.* **219**, e20211487 (2022).

36. T. Duan, Y. Du, C. Xing, H. Y. Wang, R.-F. Wang, Toll-like receptor signaling and its role in cell-mediated immunity. *Front. Immunol.* **13**, 812774 (2022).

37. S. A. Jenks, K. S. Cashman, M. C. Woodruff, F. E.-H. Lee, I. Sanz, Extrafollicular responses in humans and SLE. *Immunol. Rev.* **288**, 136–148 (2019).

38. S. A. Jenks, K. S. Cashman, E. Zumaquero, U. M. Marigorta, A. V. Patel, X. Wang, D. Tomar, M. C. Woodruff, Z. Simon, R. Bugrovsky, E. L. Blalock, C. D. Scharer, C. M. Tipton, C. Wei, S. S. Lim, M. Petri, T. B. Niewold, J. H. Anolik, G. Gibson, F. E.-H. Lee, J. M. Boss, F. E. Lund, I. Sanz, Distinct effector B cells induced by unregulated toll-like receptor 7 contribute to pathogenic responses in systemic lupus erythematosus. *Immunity* **49**, 725–739.e6 (2018).

39. S. Wang, J. Wang, V. Kumar, J. L. Karnell, B. Naiman, P. S. Gross, S. Rahman, K. Zerrouki, R. Hanna, C. Morehouse, N. Holoweckyj, H. Liu, Autoimmunity Molecular Medicine Team; Z. Manna, R. Goldbach-Mansky, S. Hasni, R. Siegel, M. Sanjuan, K. Streicher, M. P. Cancro, R. Kolbeck, R. Ettinger, IL-21 drives expansion and plasma cell differentiation of autoreactive $CD11c^{hi}T\text{-}bet^{+}$ B cells in SLE. *Nat. Commun.* **9**, 1758 (2018).

40. A. Nomura, M. Mizuno, D. Noto, A. Aoyama, T. Kuga, G. Murayama, A. Chiba, S. Miyake, Different spatial and temporal roles of monocytes and monocyte-derived cells in the pathogenesis of an imiquimod induced lupus model. *Front. Immunol.* **13**, 764557 (2022).

41. M.-L. Santiago-Raber, H. Amano, E. Amano, L. Baudino, M. Otani, Q. Lin, F. Nimmerjahn, J. S. Verbeek, J. V. Ravetch, Y. Takasaki, S. Hirose, S. Izui, Fcgamma receptor-dependent expansion of a hyperactive monocyte subset in lupus-prone mice. *Arthritis Rheum.* **60**, 2408–2417 (2009).

42. S. Kikuchi, M.-L. Santiago-Raber, H. Amano, E. Amano, L. Fossati-Jimack, T. Moll, B. L. Kotzin, S. Izui, Contribution of NZB autoimmunity 2 to Y-linked autoimmune acceleration-induced monocytosis in association with murine systemic lupus. *J. Immunol.* **176**, 3240–3247 (2006).

43. F. Mauvais-Jarvis, N. Bairey Merz, P. J. Barnes, R. D. Brinton, J.-J. Carrero, D. L. DeMeo, G. J. De Vries, C. N. Epperson, R. Govindan, S. L. Klein, A. Lonardo, P. M. Maki, L. D. McCullough, V. Regitz-Zagrosek, J. G. Regensteiner, J. B. Rubin, K. Sandberg, A. Suzuki, Sex and gender: Modifiers of health, disease, and medicine. *Lancet* **396**, 565–582 (2020).

44. A. L. Fink, K. Engle, R. L. Ursin, W.-Y. Tang, S. L. Klein, Biological sex affects vaccine efficacy and protection against influenza in mice. *Proc. Natl. Acad. Sci. U.S.A.* **115**, 12477–12482 (2018).

45. S. Oghumu, S. Varikuti, J. C. Stock, G. Volpedo, N. Saljoughian, C. A. Terrazas, A. R. Satoskar, Cutting edge: CXCR3 escapes X chromosome inactivation in T cells during infection: Potential implications for sex differences in immune responses. *J. Immunol.* **203**, 789–794 (2019).

46. A. Youness, C. Cenac, B. Faz-López, S. Grunenwald, F. J. Barrat, J. Chaumeil, J. E. Mejía, J.-C. Guéry, TLR8 escapes X chromosome inactivation in human monocytes and $CD4^+$ T cells. *Biol. Sex Differ.* **14**, 60 (2023).

47. T. Asano, B. Boisson, F. Onodi, D. Matuozzo, M. Moncada-Velez, M. R. L. Maglorius Renkilaraj, P. Zhang, L. Meertens, A. Bolze, M. Materna, S. Korniotis, A. Gervais, E. Talouarn, B. Bigio, Y. Seeleuthner, K. Bilguvar, Y. Zhang, A.-L. Neehus, M. Ogishi, S. J. Pelham, T. Le Voyer, J. Rosain, Q. Philippot, P. Soler-Palacín, R. Colobran, A. Martin-Nalda, J. G. Rivière, Y. Tandjaoui-Lambiotte, K. Chaïbi, M. Shahrooei, I. A. Darazam, N. A. Olyaei, D. Mansouri, N. Hatipoğlu, F. Palabiyik, T. Ozcelik, G. Novelli, A. Novelli, G. Casari, A. Aiuti, P. Carrera, S. Bondesan, F. Barzaghi, P. Rovere-Querini, C. Tresoldi, J. L. Franco, J. Rojas, L. F. Reyes, I. G. Bustos, A. A. Arias, G. Morelle, C. Kyheng, J. Troya, L. Planas-Serra, A. Schlüter, M. Gut, A. Pujol, L. M. Allende, C. Rodriguez-Gallego, C. Flores, O. Cabrera-Marante, D. E. Pleguezuelo, R. Pérez de Diego, S. Keles, G. Aytekin, O. Metin Akcan, Y. T. Bryceson, P. Bergman, P. Brodin, D. Smole, C. I. E. Smith, A.-C. Norlin, T. M. Campbell, L. E. Covill, L. Hammarström, Q. Pan-Hammarström, H. Abolhassani, S. Mane, N. Marr, M. Ata, F. Al Ali, T. Khan, A. N. Spaan, C. L. Dalgard, P. Bonfanti, A. Biondi, S. Tubiana, C. Burdet, R. Nussbaum, A. Kahn-Kirby, A. L. Snow, COVID Human Genetic Effort, COVID-STORM Clinicians, COVID Clinicians, Imagine COVID Group; French COVID Cohort Study Group; CoV-Contact Cohort, Amsterdam UMC Covid-19 Biobank; NIAID-USUHS COVID Study Group; J. Bustamante, A. Puel, S. Boisson-Dupuis, S.-Y. Zhang, V. Béziat, R. P. Lifton, P. Bastard, L. D. Notarangelo, L. Abel, H. C. Su, E. Jouanguy, A.

Amara, V. Soumelis, A. Cobat, Q. Zhang, J.-L. Casanova, X-linked recessive TLR7 deficiency in ~1% of men under 60 years old with life-threatening COVID-19. *Sci. Immunol.* **6**, eabl4348 (2021).

48. C. I. van der Made, A. Simons, J. Schuurs-Hoeijmakers, G. van den Heuvel, T. Mantere, S. Kersten, R. C. van Deuren, M. Steehouwer, S. V. van Reijmersdal, M. Jaeger, T. Hofste, G. Astuti, J. Corominas Galbany, V. van der Schoot, H. van der Hoeven, Wanda Hagmolen Of Ten Have; E. Klijn, C. van den Meer, J. Fiddelaers, Q. de Mast, C. P. Bleeker-Rovers, L. A. B. Joosten, H. G. Yntema, C. Gilissen, M. Nelen, J. W. M. van der Meer, H. G. Brunner, M. G. Netea, F. L. van de Veerdonk, A. Hoischen, Presence of genetic variants among young men with severe COVID-19. *JAMA* **324**, 663–673 (2020).

49. C. Fallerini, S. Daga, S. Mantovani, E. Benetti, N. Picchiotti, D. Francisci, F. Paciosi, E. Schiaroli, M. Baldassarri, F. Fava, M. Palmieri, S. Ludovisi, F. Castelli, E. Quiros-Roldan, M. Vaghi, S. Rusconi, M. Siano, M. Bandini, O. Spiga, K. Capitani, S. Furini, F. Mari, GEN-COVID Multicenter Study; A. Renieri, M. U. Mondelli, E. Frullanti, Association of toll-like receptor 7 variants with life-threatening COVID-19 disease in males: Findings from a nested case-control study. *eLife* **10**, e67569 (2021).

50. C. A. Odhams, A. L. Roberts, S. K. Vester, C. S. T. Duarte, C. T. Beales, A. J. Clarke, S. Lindinger, S. J. Daffern, A. Zito, L. Chen, L. L. Jones, L. Boteva, D. L. Morris, K. S. Small, M. M. A. Fernando, D. S. Cunninghame Graham, T. J. Vyse, Interferon inducible X-linked gene CXorf21 may contribute to sexual dimorphism in systemic lupus erythematosus. *Nat. Commun.* **10**, 2164 (2019).

51. C. Soni, O. A. Perez, W. N. Voss, J. N. Pucella, L. Serpas, J. Mehl, K. L. Ching, J. Goike, G. Georgiou, G. C. Ippolito, V. Sisirak, B. Reizis, Plasmacytoid dendritic cells and type I interferon promote extrafollicular B cell responses to extracellular self-DNA. *Immunity* **52**, 1022–1038.e7 (2020).

52. C. Soni, E. B. Wong, P. P. Domeier, T. N. Khan, T. Satoh, S. Akira, Z. S. M. Rahman, B cell-intrinsic TLR7 signaling is essential for the development of spontaneous germinal centers. *J. Immunol.* ***1950*** **193**, 4400–4414 (2014).

53. M. Manni, S. Gupta, E. Ricker, Y. Chinenov, S. H. Park, M. Shi, T. Pannellini, R. Jessberger, L. B. Ivashkiv, A. B. Pernis, Regulation of age-associated B cells by IRF5 in systemic autoimmunity. *Nat. Immunol.* **19**, 407–419 (2018).

54. E. Ricker, M. Manni, D. Flores-Castro, D. Jenkins, S. Gupta, J. Rivera-Correa, W. Meng, A. M. Rosenfeld, T. Pannellini, M. Bachu, Y. Chinenov, P. K. Sculco, R. Jessberger, E. T. L. Prak, A. B. Pernis, Altered function and differentiation of age-associated B cells contribute to the female bias in lupus mice. *Nat. Commun.* **12**, 4813 (2021).

55. F. Dossin, I. Pinheiro, J. J. Żylicz, J. Roensch, S. Collombet, A. Le Saux, T. Chelmicki, M. Attia, V. Kapoor, Y. Zhan, F. Dingli, D. Loew, T. Mercher, J. Dekker, E. Heard, SPEN integrates transcriptional and epigenetic control of X-inactivation. *Nature* **578**, 455–460 (2020).

56. W. McAlpine, L. Sun, K.-W. Wang, A. Liu, R. Jain, M. San Miguel, J. Wang, Z. Zhang, B. Hayse, S. G. McAlpine, J. H. Choi, X. Zhong, S. Ludwig, J. Russell, X. Zhan, M. Choi, X. Li, M. Tang, E. M. Y. Moresco, B. Beutler, E. Turer, Excessive endosomal TLR signaling causes inflammatory disease in mice with defective SMCR8-WDR41-C9ORF72 complex function. *Proc. Natl. Acad. Sci. U.S.A.* **115**, E11523–E11531 (2018).

57. N. Jiwrajka, N. E. Toothacre, Z. T. Beethem, S. Sting, K. S. Forsyth, A. H. Dubin, A. Driscoll, W. Stohl, M. C. Anguera, Impaired dynamic X-chromosome inactivation maintenance in T cells is a feature of spontaneous murine SLE that is exacerbated in female-biased models. *J. Autoimmun.* **139**, 103084 (2023).

58. R. Ridings-Figueroa, E. R. Stewart, T. B. Nesterova, H. Coker, G. Pintacuda, J. Godwin, R. Wilson, A. Haslam, F. Lilley, R. Ruigrok, S. A. Bageghni, G. Albadrani, W. Mansfield, J.-A. Roulson, N. Brockdorff, J. F. X. Ainscough, D. Coverley, The nuclear matrix protein CIZ1 facilitates localization of Xist RNA to the inactive X-chromosome territory. *Genes Dev.* **31**, 876–888 (2017).

59. J. D. Crawford, H. Wang, D. Trejo-Zambrano, R. Cimbro, C. C. Talbot, M. A. Thomas, A. M. Curran, A. A. Girgis, J. T. Schroeder, A. Fava, D. W. Goldman, M. Petri, A. Rosen, B. Antiochos, E. Darrah, The XIST lncRNA is a sex-specific reservoir of TLR7 ligands in SLE. *JCI Insight* **8**, e169344 (2023).

60. D. R. Dou, Y. Zhao, J. A. Belk, Y. Zhao, K. M. Casey, D. C. Chen, R. Li, B. Yu, S. Srinivasan, B. T. Abe, K. Kraft, C. Hellström, R. Sjöberg, S. Chang, A. Feng, D. W. Goldman, A. A. Shah, M. Petri, L. S. Chung, D. F. Fiorentino, E. K. Lundberg, A. Wutz, P. J. Utz, H. Y. Chang, Xist ribonucleoproteins promote female sex-biased autoimmunity. *Cell* **187**, 733–749.e16 (2024).

61. D. Djeghloul, K. Kuranda, I. Kuzniak, D. Barbieri, I. Naguibneva, C. Choisy, J.-C. Bories, C. Dosquet, M. Pla, V. Vanneaux, G. Socié, F. Porteu, D. Garrick, M. Goodhardt, Age-associated decrease of the histone methyltransferase SUV39H1 in HSC perturbs heterochromatin and B lymphoid differentiation. *Stem Cell Rep.* **6**, 970–984 (2016).

62. Y. Liu, L. Sinke, T. H. Jonkman, R. C. Slieker, BIOS Consortium; E. W. van Zwet, L. Daxinger, B. T. Heijmans, The inactive X chromosome accumulates widespread epigenetic variability with age. *Clin. Epigenetics* **15**, 135 (2023).

63. M. D. Denkinger, H. Leins, R. Schirmbeck, M. C. Florian, H. Geiger, HSC aging and senescent immune remodeling. *Trends Immunol.* **36**, 815–824 (2015).

64. M. Krasselt, C. Baerwald, Sex, symptom severity, and quality of life in rheumatology. *Clin Rev Allergy Immunol* **56**, 346–361 (2019).

65. Y. Wang, A. Roussel-Queval, L. Chasson, N. Hanna Kazazian, L. Marcadet, A. Nezos, M. H. Sieweke, C. Mavragani, L. Alexopoulou, TLR7 signaling drives the development of Sjögren's syndrome. *Front. Immunol.* **12**, 676010 (2021).

66. J. L. M. Björkegren, A. J. Lusis, Atherosclerosis: Recent developments. *Cell* **185**, 1630–1645 (2022).

67. E. J. Márquez, C. Chung, R. Marches, R. J. Rossi, D. Nehar-Belaid, A. Eroglu, D. J. Mellert, G. A. Kuchel, J. Banchereau, D. Ucar, Sexual-dimorphism in human immune system aging. *Nat. Commun.* **11**, 751 (2020).

68. E. Debrand, C. Chureau, D. Arnaud, P. Avner, E. Heard, Functional analysis of the *DXPas34* locus, a 3′ regulator of *Xist* expression. *Mol. Cell. Biol.* **19**, 8513–8525 (1999).

69. Y. Itoh, L. C. Golden, N. Itoh, M. A. Matsukawa, E. Ren, V. Tse, A. P. Arnold, R. R. Voskuhl, The X-linked histone demethylase Kdm6a in $CD4^+$ T lymphocytes modulates autoimmunity. *J. Clin. Invest.* **129**, 3852–3863 (2019).

70. C. M. Syrett, B. Paneru, D. Sandoval-Heglund, J. Wang, S. Banerjee, V. Sindhava, E. M. Behrens, M. Atchison, M. C. Anguera, Altered X-chromosome inactivation in T cells may promote sex-biased autoimmune diseases. *JCI Insight* **4**, 126751 (2019).

71. O. Demaria, P. P. Pagni, S. Traub, A. de Gassart, N. Branzk, A. J. Murphy, D. M. Valenzuela, G. D. Yancopoulos, R. A. Flavell, L. Alexopoulou, TLR8 deficiency leads to autoimmunity in mice. *J. Clin. Invest.* **120**, 3651–3662 (2010).

72. C. Libert, L. Dejager, I. Pinheiro, The X chromosome in immune functions: When a chromosome makes the difference. *Nat. Rev. Immunol.* **10**, 594–604 (2010).

73. S. Becker-Herman, A. Meyer-Bahlburg, M. A. Schwartz, S. W. Jackson, K. L. Hudkins, C. Liu, B. D. Sather, S. Khim, D. Liggitt, W. Song, G. J. Silverman, C. E. Alpers, D. J. Rawlings, WASp-deficient B cells play a critical, cell-intrinsic role in triggering autoimmunity. *J. Exp. Med.* **208**, 2033–2042 (2011).

74. A. Mohr, M. Atif, R. Balderas, G. Gorochov, M. Miyara, The role of FOXP3$^+$ regulatory T cells in human autoimmune and inflammatory diseases. *Clin. Exp. Immunol.* **197**, 24–35 (2019).

75. H.-H. Yu, Y.-H. Yang, B.-L. Chiang, Chronic granulomatous disease: A comprehensive review. *Clin Rev Allergy Immunol* **61**, 101–113 (2021).

76. K. Borziak, J. Finkelstein, X-linked genetic risk factors that promote autoimmunity and dampen remyelination are associated with multiple sclerosis susceptibility. *Mult. Scler. Relat. Disord.* **66**, 104065 (2022).

77. X. Lian, R. Xiao, X. Hu, T. Kanekura, H. Jiang, Y. Li, Y. Wang, Y. Yang, M. Zhao, Q. Lu, DNA demethylation of CD40l in CD4$^+$ T cells from women with systemic sclerosis: A possible explanation for female susceptibility. *Arthritis Rheum.* **64**, 2338–2345 (2012).

78. Y. Liu, J. Liao, M. Zhao, H. Wu, S. Yung, T. M. Chan, A. Yoshimura, Q. Lu, Increased expression of TLR2 in CD4$^+$ T cells from SLE patients enhances immune reactivity and promotes IL-17 expression through histone modifications. *Eur. J. Immunol.* **45**, 2683–2693 (2015).

79. L.-C. Su, W.-D. Xu, A.-F. Huang, IRAK family in inflammatory autoimmune diseases. *Autoimmun. Rev.* **19**, 102461 (2020).

80. A. A. de Jesus, Y. Hou, S. Brooks, L. Malle, A. Biancotto, Y. Huang, K. R. Calvo, B. Marrero, S. Moir, A. J. Oler, Z. Deng, G. A. Montealegre Sanchez, A. Ahmed, E. Allenspach, B. Arabshahi, E. Behrens, S. Benseler, L. Bezrodnik, S. Bout-Tabaku, A. C. Brescia, D. Brown, J. M. Burnham, M. S. Caldirola, R. Carrasco, A. Y. Chan, R. Cimaz, P. Dancey, J. Dare, M. DeGuzman, V. Dimitriades, I. Ferguson, P. Ferguson, L. Finn, M. Gattorno, A. A. Grom, E. P. Hanson, P. J. Hashkes, C. M. Hedrich, R. Herzog, G. Horneff, R. Jerath, E. Kessler, H. Kim, D. J. Kingsbury, R. M. Laxer, P. Y. Lee, M. A. Lee-Kirsch, L. Lewandowski, S. Li, V. Lilleby, V. Mammadova, L. N. Moorthy, G. Nasrullayeva, K. M. O'Neil, K. Onel, S. Ozen, N. Pan, P. Pillet, D. G. Piotto, M. G. Punaro, A. Reiff, A. Reinhardt, L. G. Rider, R. Rivas-Chacon, T. Ronis, A. Rösen-Wolff, J. Roth, N. M. Ruth, M. Rygg, H. Schmeling, G. Schulert, C. Scott, G. Seminario, A. Shulman, V. Sivaraman, M. B. Son, Y. Stepanovskiy, E. Stringer, S. Taber, M. T. Terreri, C. Tifft, T. Torgerson, L. Tosi, A. Van Royen-Kerkhof, T. Wampler Muskardin, S. W. Canna, R. Goldbach-Mansky, Distinct interferon signatures and cytokine patterns define additional systemic autoinflammatory diseases. *J. Clin. Invest.* **130**, 1669–1682 (2020).

81. Y. Lee, A. W. Wessel, J. Xu, J. G. Reinke, E. Lee, S. M. Kim, A. P. Hsu, J. Zilberman-Rudenko, S. Cao, C. Enos, S. R. Brooks, Z. Deng, B. Lin, A. A. de Jesus, D. N. Hupalo, D. G. Piotto, M. T. Terreri, V. R. Dimitriades, C. L. Dalgard, S. M. Holland, R. Goldbach-Mansky, R. M. Siegel, E. P. Hanson, Genetically programmed alternative splicing of NEMO mediates an autoinflammatory disease phenotype. *J. Clin. Invest.* **132**, e128808 (2022).

82. X.-P. Ye, F.-F. Yuan, L.-L. Zhang, Y.-R. Ma, M.-M. Zhang, W. Liu, F. Sun, J. Wu, M. Lu, L.-Q. Xue, J.-Y. Shi, S.-X. Zhao, H.-D. Song, J. Liang, C.-X. Zheng, ITM2A expands evidence for genetic and environmental interaction in graves disease pathogenesis. *J. Clin. Endocrinol. Metab.* **102**, 652–660 (2017).

83. C. McDonald, C. Xanthopoulos, E. Kostareli, The role of Bruton's tyrosine kinase in the immune system and disease. *Immunology* **164**, 722–736 (2021).

84. J. Zhang, Y. Zhang, J. Yang, L. Zhang, L. Sun, H.-F. Pan, N. Hirankarn, D. Ying, S. Zeng, T. L. Lee, C. S. Lau, T. M. Chan, A. M. H. Leung, C. C. Mok, S. N. Wong, K. W. Lee, M. H. K. Ho, P. P. W. Lee,

B. H.-Y. Chung, C. Y. Chong, R. W. S. Wong, M. Y. Mok, W. H. S. Wong, K. L. Tong, N. K. C. Tse, X.-P. Li, Y. Avihingsanon, P. Rianthavorn, T. Deekajorndej, K. Suphapeetiporn, V. Shotelersuk, S. K. Y. Ying, S. K. S. Fung, W. M. Lai, M.-M. Garcia-Barceló, S. S. Cherny, P. K.-H. Tam, Y. Cui, P. C. Sham, S. Yang, D. Q. Ye, X.-J. Zhang, Y. L. Lau, W. Yang, Three SNPs in chromosome 11q23.3 are independently associated with systemic lupus erythematosus in Asians. *Hum. Mol. Genet.* **23**, 524–533 (2014).
